# Development and Optimization of a Lupeol-Loaded Bilosomal Gel for Enhanced Topical Therapy of Imiquimod-Induced Psoriasis

**DOI:** 10.3390/gels12100918

**Published:** 2026-10-13

**Authors:** Mohammad Hamza, Soumya Samanta, Mohammed Faheem Khan, Supriya Roy, Zeeshan Fatima, Ritu Gupta, Salvatore Gallo, Nimisha Srivastava

**Affiliations:** 1 Department of Pharmaceutics, Amity Institute of Pharmacy, Amity University Uttar Pradesh, Lucknow Campus, Lucknow 226028, India; mohd.hamza4577@gmail.com (M.H.);; 2 Chemistry Division, Department of Biotechnology, Era University, Lucknow 226003, India; 3 Department of Pharmacology, Amity Institute of Pharmacy, Amity University Uttar Pradesh, Lucknow Campus, Lucknow 226028, India; 4 Department of Chemistry, Amity Institute of Pharmacy, Amity University Uttar Pradesh, Lucknow Campus, Lucknow 226028, India; 5 Faculty of Pharmaceutical Science, University of British Columbia, Vancouver, BC V5V0A8, Canada; connect2rgupta@gmail.com; 6 Department of Physics and Astronomy “E. Maiorana”, University of Catania, 95123 Catania, Italy

**Keywords:** anti-inflammatory, Box–Behnken Design (BBD), lupeol, psoriasis, topical delivery

## Abstract

Lupeol, a naturally occurring pentacyclic triterpenoid, exhibits potent anti-inflammatory and antioxidant activities, making it a promising candidate for psoriasis treatment. However, its poor aqueous solubility and limited skin permeation restrict its therapeutic application. This study aimed to develop and optimize a lupeol-loaded bilosomal gel to enhance topical delivery and improve anti-psoriatic efficacy. Lupeol-loaded bilosomes were prepared using the thin-film hydration method and optimized through a Box–Behnken experimental design. The optimized formulation was characterized for vesicle size, entrapment efficiency, drug release, and physicochemical properties before incorporation into a Carbopol^®^ 934P gel. The bilosomal gel was further evaluated through in vitro drug release, ex vivo skin permeation, and in vivo efficacy in an imiquimod-induced psoriasis-like mice model. The optimized bilosomes exhibited nanoscale vesicles, high drug entrapment, and sustained drug release. Incorporation into the gel produced a formulation with suitable physicochemical characteristics and significantly enhanced skin permeation compared with a conventional gel. In vivo, the bilosomal gel markedly reduced Psoriasis Area and Severity Index (PASI) scores, spleen index, and TNF-α levels while restoring normal skin histology. These findings demonstrate that bilosomal encapsulation substantially improves the topical delivery and therapeutic efficacy of lupeol, highlighting the developed bilosomal gel as a promising nanocarrier-based strategy for the topical management of psoriasis.

## 1. Introduction

Psoriasis is a chronic, immune-mediated inflammatory skin disorder characterized by epidermal hyperproliferation, abnormal keratinocyte differentiation, and persistent inflammation. Clinically, it can be classified into different types, including plaque, flexural, guttate, pustular, or erythrodermic psoriasis. Globally, it has affected nearly 60 million people, which accounts for around 2% of the population [1]. This disease significantly affects individuals physically and psychologically. The pathogenesis of psoriasis is complex and multifactorial, involving genetic susceptibility and environmental triggers. Current studies have highlighted the crucial role of skin-resident immune cells, major signal transduction pathways, and pro-inflammatory cytokines such as tumor necrosis factor (TNF)-α, interleukin (IL)-23p19, and IL-17A for the treatment of psoriasis [2]. Although several synthetic agents and biologics have been used to target these cytokines, their long-term use is associated with adverse effects, high costs and systemic toxicity. Due to these limitations, natural therapeutics are being progressed for the treatment of skin disorders. These are safer and more effective than synthetic compounds [3].

Among the naturally derived bioactive compounds, lupeol, a pentacyclic triterpenoid widely distributed in fruits and vegetables, has gained considerable attention for the management of inflammatory skin disorders such as psoriasis. It possesses multiple pharmacological properties, including antioxidant, wound healing, anti-inflammatory, antihyperglycemic, anti-arthritic, hepatoprotective and antimicrobial properties. Lupeol also has the ability to stimulate the proliferation of skin cells and promote their migration, thereby repairing the damaged skin. These properties make lupeol a promising therapeutic candidate for topical psoriasis treatment [4,5,6].

However, despite its significant therapeutic potential, the application of lupeol is limited due to its unfavorable physicochemical properties. Lupeol is classified as a Biopharmaceutics Classification System (BCS) class II drug. It is characterized by high lipophilicity and poor aqueous solubility, which limits its permeability across the stratum corneum [7,8]. Several conventional topical formulations such as creams, ointments, gels and emulsions have been explored to deliver lupeol, but these systems often suffer from poor skin permeation and poor drug retention at the target site, low drug loading, and poor stability in biological environments. These limitations indicate that an ideal topical carrier for lupeol should simultaneously improve drug solubility, vesicle stability, skin permeation, and local drug retention while minimizing systemic exposure. Such requirements remain insufficiently addressed by currently available lupeol formulations, highlighting the need for an advanced vesicular delivery platform [9,10].

To overcome these limitations, bilosomes, a novel lipid-based vesicular drug delivery system, have emerged as a promising strategy for topical drug delivery. Bilosomes are composed of cholesterol and non-ionic surfactants with bile salts incorporated into the bilayer structure [11]. Conacher et al. (2001) first introduced bilosomes as bile salt-stabilized vesicular systems to improve oral drug delivery by enhancing stability and permeation [12]. Incorporation of bile salts enhances membrane fluidity, increases vesicular deformability, improves the transportation of drugs through biological membranes and enhances the solubility of poorly water-soluble compounds. Compared to conventional vesicular systems, bilosomes offer several advantages such as high drug loading capacity, improved stability, reduced toxicity, and controlled drug release [11]. Recent studies have demonstrated the potential of bilosomes in transdermal and topical drug delivery. Abdelbari et al. (2023) developed Brij^®^-integrated bilosomes to enhance transdermal delivery of niflumic acid for the treatment of osteoarthritis, reporting improved permeation and therapeutic efficacy [13]. In the treatment of psoriasis, bilosomes not only act as efficient drug carriers but also as localizers, retaining higher drug concentrations at the diseased site and minimizing systemic exposure [11].

Despite the potential efficacy of bilosomes in transdermal and topical drug delivery, further investigation is needed to explore their application for the topical delivery of lupeol in psoriasis. The present study therefore focused on the development and optimization of a lupeol-loaded bilosomal gel using a Quality-by-Design (QbD) approach, followed by evaluation of its physicochemical characteristics, ex vivo skin permeation, and anti-psoriatic efficacy in an imiquimod-induced psoriasis model. Most reported lupeol formulations, including conventional gels, nanoemulsions, liposomes, and solid lipid nanoparticles, primarily focused on improving solubility or drug release, but limited attention has been given to simultaneously enhancing vesicle stability, deformability, localized skin retention, and anti-psoriatic efficacy. Therefore, the lupeol-loaded bilosomal gel offers a novel strategy for localized psoriasis management [14,15,16,17,18].

Based on the above knowledge gap, the present study was designed to develop and optimize a lupeol-loaded bilosomal gel as an advanced topical delivery system for the management of psoriasis. A Box–Behnken Design (BBD) was employed to optimize critical formulation variables and to investigate their influence on vesicle size, entrapment efficiency, and drug release. The optimized bilosomes were subsequently incorporated into a Carbopol 934P gel and comprehensively characterized for physicochemical properties, ex vivo skin permeation, dermato-kinetics, and in vivo anti-psoriatic efficacy in an imiquimod-induced psoriasis Swiss albino mouse model, with comparison against a conventional lupeol gel and the standard drug tacrolimus.

## 2. Results and Discussion

The present study was designed to develop and evaluate a lupeol-loaded bilosomal gel for enhanced topical delivery and improved therapeutic efficacy in the management of psoriasis.

### 2.1. High-Performance Liquid Chromatography (HPLC) Analysis Result

**Figure 1:**
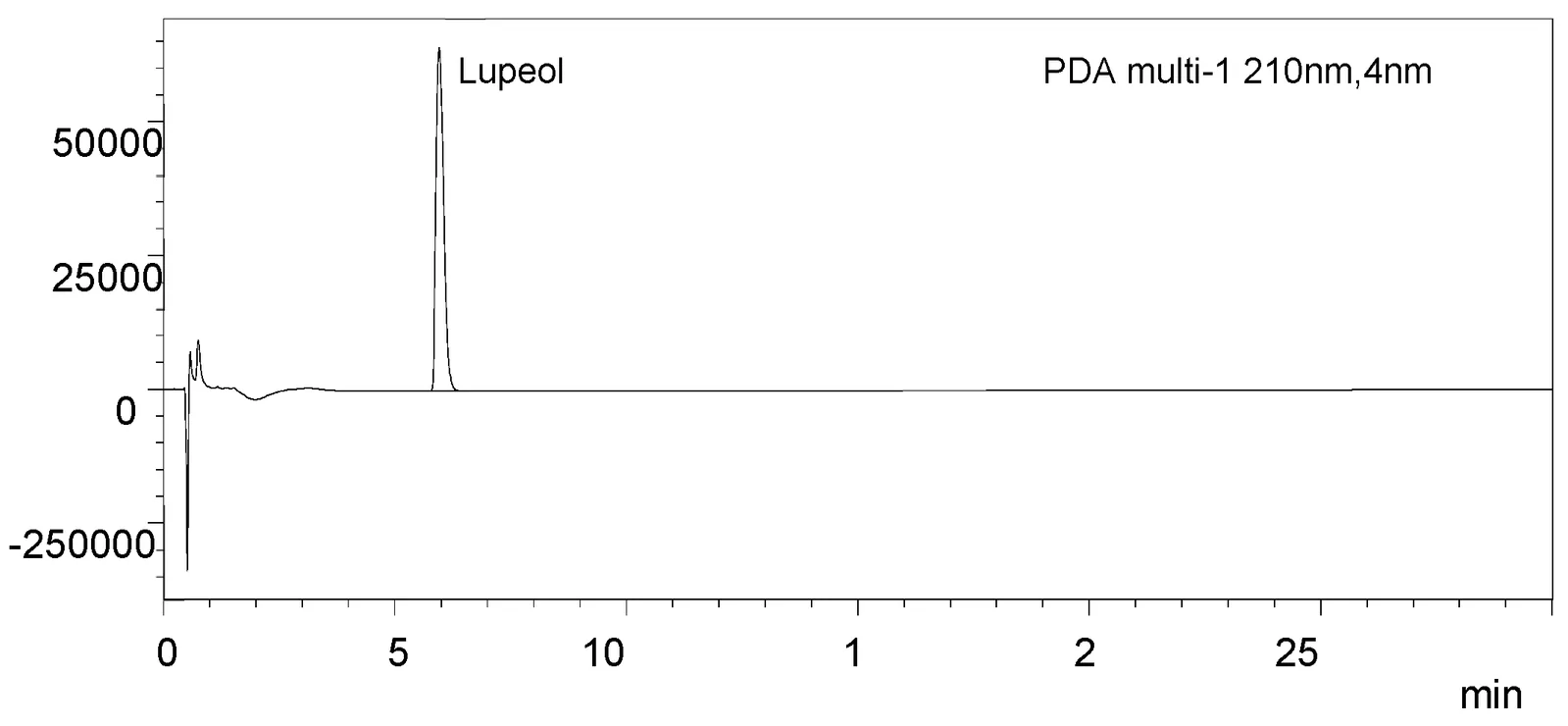
The HPLC chromatogram of the drug sample, exhibiting a characteristic peak at the retention time corresponding to lupeol.

The drug sample was identified using High-Performance Liquid Chromatography (HPLC). The sample was found to be lupeol (Figure 1). The HPLC chromatogram of the sample exhibited a single, sharp peak at a retention time of 5.96 min. The retention time was consistent with the standard reference sample, which confirmed the presence of lupeol in the sample. A similar chromatographic study was done by Jayaprakasam et al. [19].

### 2.2. Drug–Excipient Interaction Study

**Figure 2:**
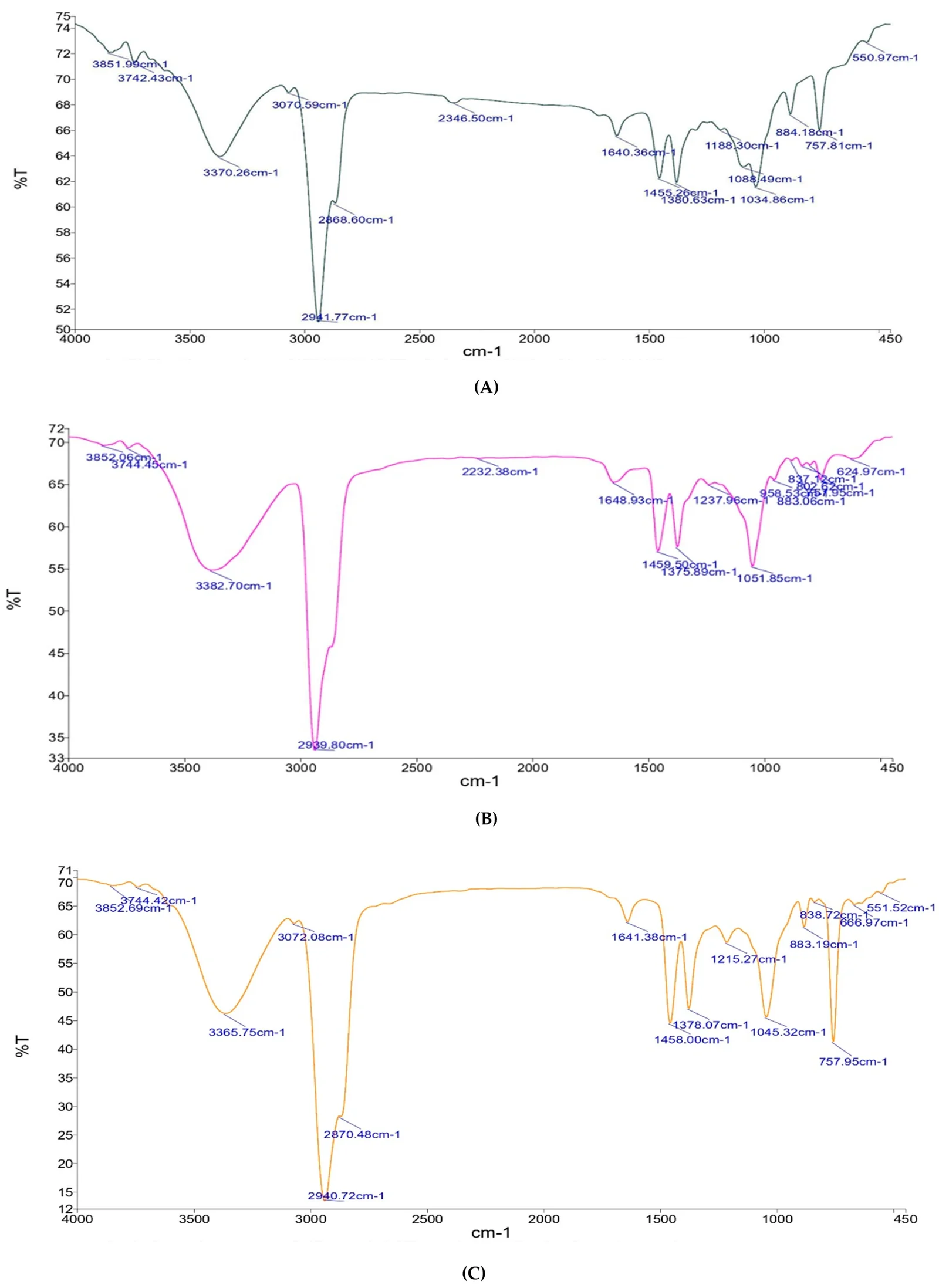
FTIR images for excipient compatibility check: (**A**) pure lupeol, (**B**) cholesterol, (**C**) lyophilized optimized LUPB.

The FTIR spectra of lupeol, cholesterol, and the lyophilized optimized formulation are presented in Figure 2. Upon comparison of the spectra, the characteristic peaks of lupeol and cholesterol were retained in the lyophilized formulation, with only minor changes in their peak positions. No significant disappearance of characteristic peaks or appearance of new peaks was observed. This suggests that there were no discernible chemical interactions between lupeol and the formulation excipients. The observed changes in the peak positions and intensities in the formulation may be attributed to the incorporation of lupeol within the formulation matrix.

### 2.3. Optimization of Lupeol-Loaded Bilosomes (LUPB)

Lupeol-loaded bilosomes (LUPB) was prepared by thin-film hydration method and was optimized by the Box–Behnken Design (BBD) method. The BBD layout and three measured responses—vesicle size (nm), entrapment efficiency (%) and drug release (%)—are recorded in Table 1. Design-Expert^®^ version 13 was used to sequentially fit polynomial models (Linear, 2FI, and Quadratic) to all three responses using model comparison statistics (Table 2) and least-squares regression (Table 3). Models were evaluated using ANOVA (Table 4), lack of fit, and Akaike Information Criterion (AICc) (Table 2), with the highest-order significant model having the lowest AICc and preserving the model hierarchy selected for further analysis.

#### 2.3.1. Effect of Factors (Independent Variables) on Vesicle Size

Using Design-Expert^®^ (version 13.0), linear, 2FI, and quadratic models were sequentially fitted, and predicted vesicle sizes were derived from the selected quadratic polynomial model. As shown by ANOVA (Table 4), the model was highly significant (*p* < 0.0001), indicating only a 0.01% chance that the observed model significance resulted from random noise. The calculated model F-value (321.63) greatly exceeded the critical F-value (4.459), confirming the model’s overall significance. A non-significant lack of fit (*p* = 0.1742; F = 4.98) indicated that the model adequately fits the experimental data.

The polynomial equation obtained for vesicle size is shown by Equation (1):

(1) Vesicle size (Y_1_) = +286.00 + 11.23A − 9.77B − 31.50C − 30.80AB − 50.75AC − 0.75BC − 9.15A^2^ − 25.15B^2^ + 19.90C^2^

Across the 15 Box–Behnken design runs, the hydrodynamic vesicle size of lupeol-loaded bilosomes varied from 217 ± 0.23 to 390 ± 0.21 nm, with PDI values below 0.5 (Table 1). Bilosomes form nanosized vesicles with a high surface-area-to-volume ratio, which may facilitate close contact with the stratum corneum. Combined with the fluidizing and permeation-enhancing effects of the incorporated bile salts, their nanosized dimensions may facilitate penetration into deeper skin layers, thereby supporting effective transdermal drug delivery [20]. Polynomial Equation (1) shows that cholesterol (A), Span 60 concentration (B), and sonication time (C) significantly affect vesicle formation, with cholesterol showing a positive effect (+11.23), while Span 60 concentration (−9.77) and sonication time (−31.50) show negative effects. This trend was generally reflected in the experimental data. At a low Span 60 concentration (50%), increasing cholesterol from 10 to 30 mg increased vesicle size from 217 nm (F1) to 305 nm (F2), confirming the vesicle-enlarging effect of cholesterol, which may be attributed to increased membrane rigidity and bilayer thickness [21]. Similarly, at 60% Span 60 and 10 min of sonication, vesicle size increased markedly from 270 nm (F5) to 390 nm (F6) with increasing cholesterol. Consistent with our findings, Elkomy et al. reported increased vesicle size with higher cholesterol levels in berberine-loaded bilosomes, which was attributed to greater dispersion and intercalation of cholesterol molecules within the bilosome vesicles [22]. Needham et al. reported that increasing cholesterol enhanced vesicle cohesion and membrane stiffness, thereby affecting vesicle formation and stability [23]. Hung et al. showed that cholesterol condensed the lipid layer while increasing membrane thickness. Ahmed et al. similarly reported larger lornoxicam-loaded bilosomes with increasing lipid concentration [24].

Sonication time (with linear effect coefficient −31.50) emerged as the most dominant factor for size reduction by supplying shear stress to disrupt larger aggregates. The sonication process generates intense acoustic cavitation within the dispersion medium, which fragmented larger vesicles into smaller ones until a stable nanosized population was obtained. This explains the observed decrease in vesicle size with increasing sonication time [25]. El-Nabarawi et al. also demonstrated that sonication time has a significant negative (inverse) influence on daclatasvir-loaded bilosome vesicle size; prolonging the duration directly decreases vesicle diameter [26]. In agreement with our findings, Al-Edhari and Al-Gawhari reported that longer sonication reduced nisoldipine bilosome size through acoustic cavitation, where ultrasonic waves generate and collapse microbubbles to disrupt vesicles [25]. This trend changed at higher Span 60 concentration and longer sonication. For example, under identical surfactant (Span 60) concentration (60%) and sonication time (20 min), F8 (30 mg cholesterol) exhibited a markedly smaller vesicle size (222 nm) than F7 (10 mg cholesterol, (305 nm), indicating a significant interaction between cholesterol and sonication predicted by the negative AC coefficient.

**Figure 3:**
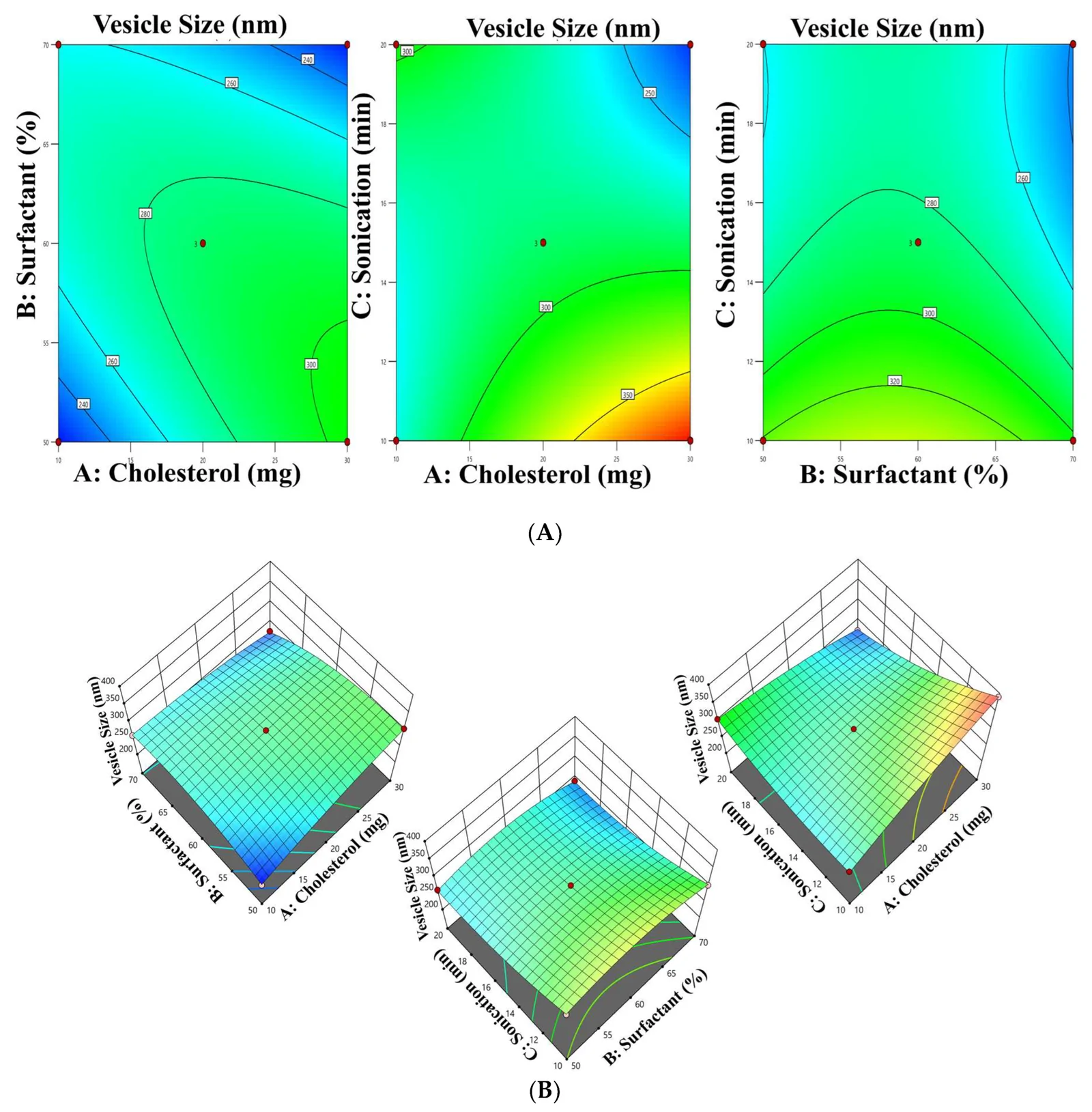
(**A**) The contour plots representing the effect of cholesterol, surfactant (Span 60) concentration and sonication time on vesicle size of lupeol-loaded bilosomes. (**B**) The three-dimensional response surface plots illustrating the effect of independent variables on the vesicle size of lupeol-loaded bilosomes.

Increasing Span 60 concentration and extending sonication generally produced smaller vesicles by lowering interfacial tension and providing sufficient ultrasonic energy to break larger vesicles into nanosized particles [27]. Khafagy ES et al. reported that higher surfactant (Span 60) concentration reduced vesicle size in simvastatin-loaded bilosomes developed for enhanced transdermal delivery. This effect was attributed to reduced interfacial tension) [28]. Likewise, in sildenafil citrate-loaded bilosomes, higher Span 60 concentrations promoted smaller vesicle formation by increasing membrane curvature [29]. The negative interaction terms (AB and AC) further suggest that adequate Span 60 concentration and controlled sonication effectively counterbalance the vesicle-enlarging effect of cholesterol. The center-point formulations (F13–F15) produced highly reproducible vesicle sizes (285–288 nm), indicating good experimental precision and confirming the adequacy of the quadratic model [30]. These relationships were further confirmed by Contour (2D) plots and 3D response surface plots (Figure 3A,B).

#### 2.3.2. Effects of Factors (Independent Variables) on Entrapment Efficiency

The quadratic model for entrapment efficiency was highly significant (F = 89.31, *p* < 0.0001), indicating that the selected formulation variables significantly influenced drug entrapment. The lack of fit was non-significant (F = 0.56, *p* = 0.6904), confirming that the model adequately described the experimental data and possessed good predictive capability, as shown in Table 4. The polynomial equation obtained was

(2) Entrapment efficiency (Y_2_) = +83.67 + 5.37A + 3.87B − 3.75C + 2.50AB − 2.25AC + 2.75BC − 6.58A^2^ +2.92B^2^ − 3.83C^2^

**Figure 4:**
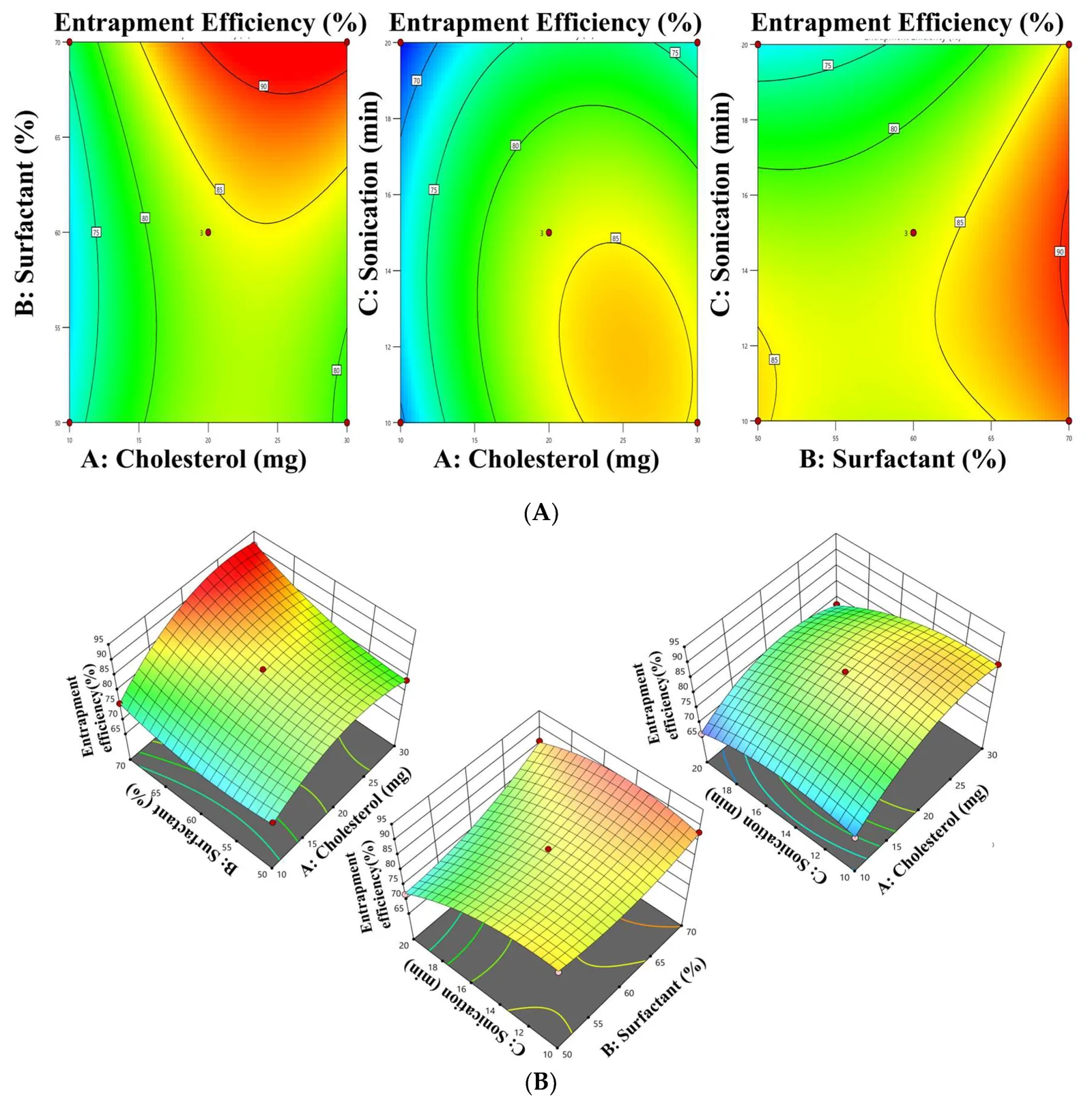
(**A**) The contour plots representing the effect of cholesterol, surfactant (Span 60) concentration and sonication time on entrapment efficiency (%) of lupeol-loaded bilosomes. (**B**) The three-dimensional (3D) response surface plots illustrating the effect of independent variables on the entrapment efficiency (%) of lupeol-loaded bilosomes.

The regression model showed that both cholesterol amount (A, +5.37) and surfactant concentration (B, +3.87) displayed positive linear impacts on drug entrapment, whereas sonication time (C, −3.75) exerted a negative impact. These trends are presented in the contour plot (2D), and 3D response surface plots are shown in Figure 4A,B.

Increasing cholesterol consistently improved drug entrapment efficiency, as evidenced by the increase in entrapment efficiency from 74% (F1) to 79% (F2) and from 69% (F5) to 85% (F6) when cholesterol increased from 10 to 30 mg. Cholesterol likely enhanced bilayer stability and reduced membrane permeability, thereby minimizing the leakage of lipophilic drug lupeol during vesicle formation. Supporting our findings, Elkomy et al. demonstrated that increasing cholesterol concentration enhanced drug entrapment in berberine-loaded bilosomes. This effect was attributed to increased lipid-bilayer hydrophobicity and stiffness, which reduced drug leakage [31]. In another study, Khafagy et al. demonstrated that increasing cholesterol concentration enhances carvedilol entrapment efficiency by stabilizing the bilosomal membrane to prevent drug leakage. Additionally, higher cholesterol levels increase the bilayer’s hydrophobicity, accommodating a greater payload of the lipophilic drug within the vesicles [32].

The increase in surfactant (Span 60) concentration enhanced entrapment efficiency by formation of stable bilosomal vesicles with improved drug incorporation within the lipid bilayer. The long alkyl chain (C18) and high phase transition temperature of the non-ionic surfactant (Span 60) increase the hydrophobicity and structural integrity of the bilosomal membrane. In addition, an adequate surfactant concentration lowers the interfacial tension during vesicle formation and reduces the drug leakage, which enhances the entrapment of drug. However, excessive surfactant concentrations may fluidize the bilayer of vesicles, which results in the partitioning of drug into the aqueous phase and a subsequent decline in entrapment efficiency [33,34,35,36]. At 30 mg cholesterol and 15 min sonication, increasing surfactant concentration from 50% to 70% increased entrapment efficiency from 79% (F2) to 91% (F4), representing the highest encapsulation among all formulations.

In contrast, increasing sonication time generally decreased entrapment efficiency. During sonication, high shear forces, shock waves, and microstreaming were generated, which progressively disrupt the integrity of the bilosome vesicles. Although moderate sonication is necessary to reduce vesicle size, excessive sonication increases membrane permeability and may induce transient defects or partial disruption of the bilayer, facilitating the diffusion of the encapsulated drug into the external medium. Consequently, prolonged sonication results in reduced drug retention and lower entrapment efficiency [21,33,37]. This phenomenon was evident in the present study, where at 10 mg cholesterol and 60% Span 60, the entrapment efficiency decreased from 69% (F5) to 66% (F7) as the sonication time increased from 10 to 20 min. The replicated center-point formulations (83–85%) demonstrated excellent reproducibility, further validating the predictive capability of the developed quadratic model.

Overall, cholesterol and surfactant (Span 60) acted synergistically to maximize drug entrapment, whereas excessive sonication time reduced encapsulation. Positive binary interactions (AB = +2.50, BC = +2.75) and negative quadratic effects (A^2^ = −6.58, C^2^ = −3.83) confirm that maximum entrapment requires careful optimization of cholesterol and surfactant levels to avoid bilayer saturation or mechanical disruption [25].

#### 2.3.3. Effects of Factors (Independent Variables) on % Drug Release

The ANOVA results demonstrated that the quadratic model for drug release was highly significant (*p* < 0.0001), confirming a significant effect of the formulation variables on drug release. The non-significant lack of fit (F = 1.35, *p* = 0.4773) indicated that no significant lack of fit was detected for the quadratic model. The percentage drug release of quadratic in coded factors is presented as the following equation:

(3) Drug release (Y_3_) = +71.00 − 7.25A + 7.87B + 3.63C + 1.50BC + 11.75A^2^ − 8.00B^2^ + 2.00C^2^

According to polynomial Equation (3), surfactant (Span 60) concentration (B, +7.87) and sonication time (C, +3.63) significantly enhanced drug release, whereas cholesterol (A, −7.25) acted as a retarding agent. With the smallest linear coefficient, surfactant (Span 60) showed the least effect among the three factors [38]. The experimental findings strongly supported these predictions. Increasing cholesterol level significantly decreased drug release [39]. Cholesterol is incorporated in bilosome vesicles which decrease the membrane fluidity and permeability. As a result, the diffusion of the encapsulated lipophilic drug from the bilosomal membrane is retarded, leading to a more sustained release profile [21,40,41]. For example, at 50% surfactant and 15 min sonication, drug release declined from 75% (F1) to 58% (F2) as cholesterol increased from 10 to 30 mg. Likewise, at 60% surfactant and 10 min sonication, increasing cholesterol decreased the drug release from 88% (F5) to 75% (F6). The large positive quadratic term for cholesterol (A^2^ = +11.75) reflects a non-linear curvature where release drops sharply at moderate cholesterol concentrations before stabilizing, demonstrating that balancing A, B, and C allows fine-tuning toward maximum drug release.

Increasing surfactant (Span 60) concentration promoted faster drug release by improving membrane fluidity and enhancing wettability. The enhanced drug release at higher surfactant concentrations may result from less organized and more permeable vesicular lipid bilayers, facilitating drug diffusion and release [42]. At low cholesterol, increasing surfactant from 50% to 70% increased lupeol release from 75% (F1) to 90% (F3)**.** Similarly, Sideek et al. demonstrated that increasing the Span^®^ 60:cholesterol ratio from 1:5 to 1:10 enhanced 72 h curcumin release, which is attributed to greater drug solubility at higher Span^®^ 60 concentrations [43].

Sonication also enhanced drug release by reducing the vesicle size through acoustic cavitation. The collapse of cavitation bubbles during ultrasonication generates localized shear forces that fragment larger vesicles into smaller and more homogeneous bilosomes. The reduction in vesicle size increases the surface area-to-volume ratio and shortens the diffusion path length for the encapsulated drug, thereby facilitating faster drug diffusion across the lipid bilayer into the release medium [21,33,44]. This was evident in formulations containing 10 mg cholesterol and 60% surfactant, where drug release increased from 88% (F5) to 95% (F7) as sonication time increased from 10 to 20 min.

**Figure 5:**
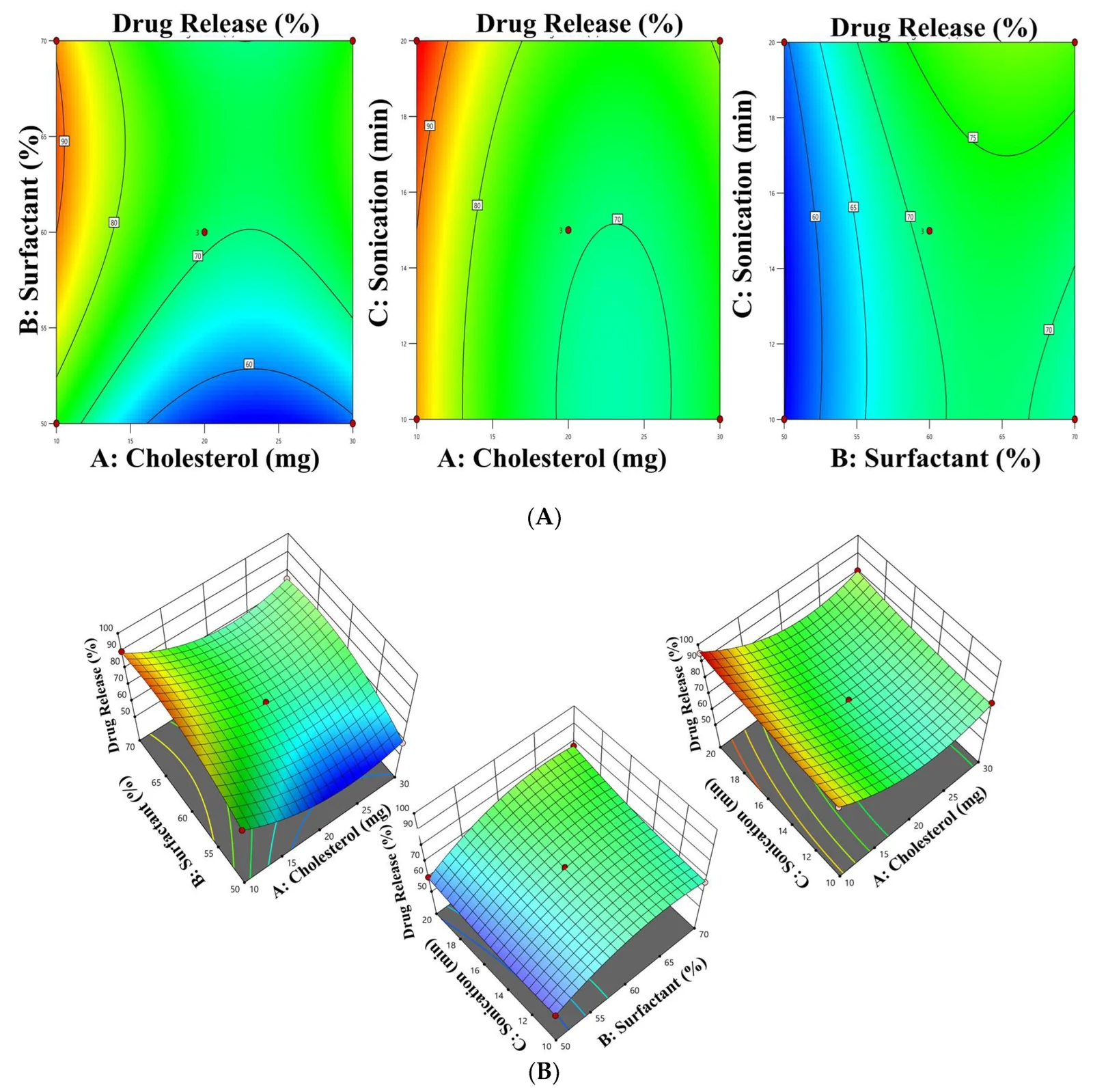
(**A**) The 2D contour plots representing the effect of cholesterol, surfactant (Span 60) concentration and sonication time on vesicle size of lupeol-loaded bilosomes. (**B**) The three-dimensional response surface plots illustrating the effect of independent variables on the drug release (%) of lupeol-loaded bilosomes.

The center-point formulations exhibited consistent release values (70–72%), confirming good experimental reproducibility. Collectively, these findings indicate that sustained drug release can be achieved through higher cholesterol levels, whereas increasing surfactant concentration and sonication time accelerates drug release by producing smaller, more permeable bilosomes. The close agreement between the experimental observations and the polynomial model demonstrates the suitability of the Box–Behnken design for optimizing bilosomal formulations [33,39]. The contour plots (2D) and three-dimensional response surface plots are presented in Figure 5A,B.

The actual-versus-predicted plots showed generally good agreement between experimental and model-predicted responses, although a greater deviation was observed for drug release at the optimized formulation.

#### 2.3.4. Optimized Lupeol-Loaded Bilosomes (LUPB) & Model Validation

The optimized bilosomal formulation (presented in Table 5) was obtained with the composition of 30 mg cholesterol, 63.535% surfactant (Span 60) and 13.344 min of sonication, with an overall desirability value (D) of 0.820. The observed (actual) responses showed close agreement with the values predicted using Design-Expert^®^ (version 13.0) software, with accuracies of 99.7%, 98.06% and 88.93% for vesicle size.

To validate the model’s predictive accuracy, three independent replicates were prepared under the optimized formulation conditions. The observed responses were compared with the predicted values against 95% prediction intervals (PIs) to confirm model reliability. The observed and predicted values showed close agreement for vesicle size and entrapment efficiency, whereas drug release showed a positive deviation from the model prediction, with prediction errors of 0.30% and 1.94%, respectively, while drug release exhibited a comparatively higher prediction error of 11.07% (Table 6).

### 2.4. Characterization of Optimized Lupeol-Loaded Bilosomes (LUPB)

**Figure 6:**
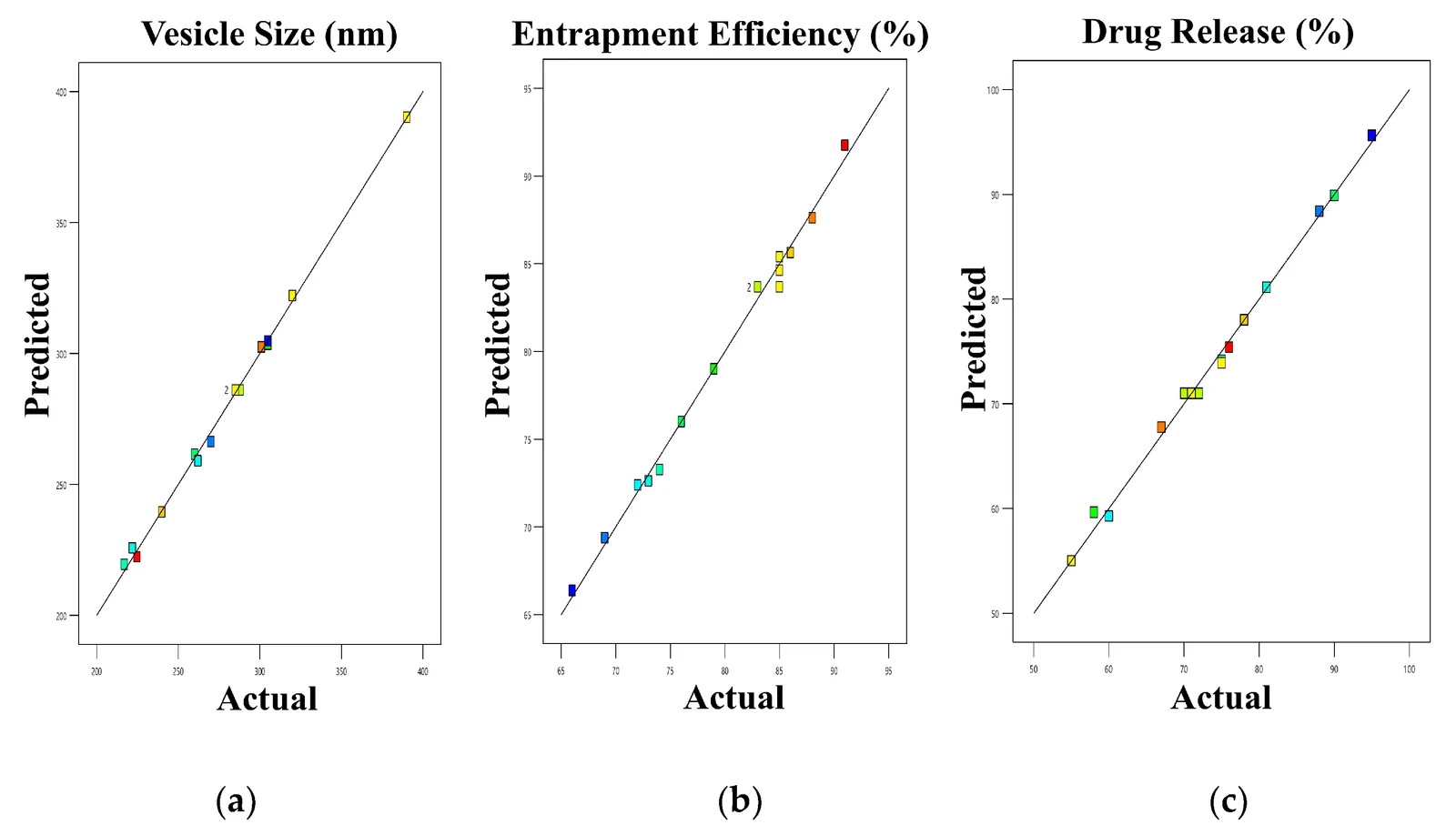
The graphs of actual and predicted values of the responses: (**a**) vesicle size, (**b**) entrapment efficiency, and (**c**) drug release.

**Figure 7:**
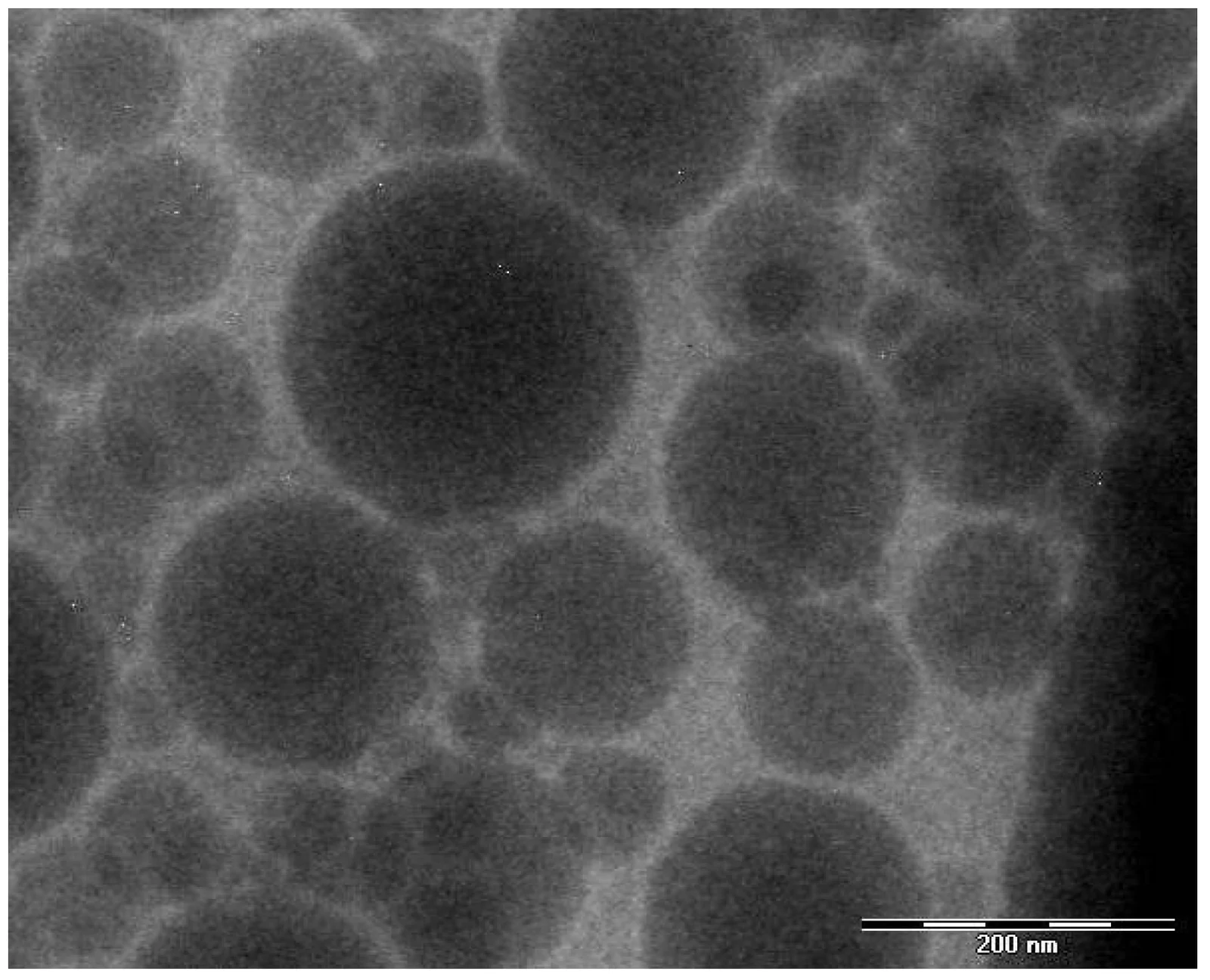
The TEM image showing the morphology of the optimized lupeol-loaded bilosomes.

The morphology of the designed bilosomes was evaluated by TEM studies (Figure 6). Representative TEM images showed the morphology of the optimized formulation. The TEM images showed spherical and possessed a smooth surface, as shown in Figure 7; however, the TEM observations were considered qualitative and were not used to establish the absence of aggregation throughout the formulation.

**Figure 8:**
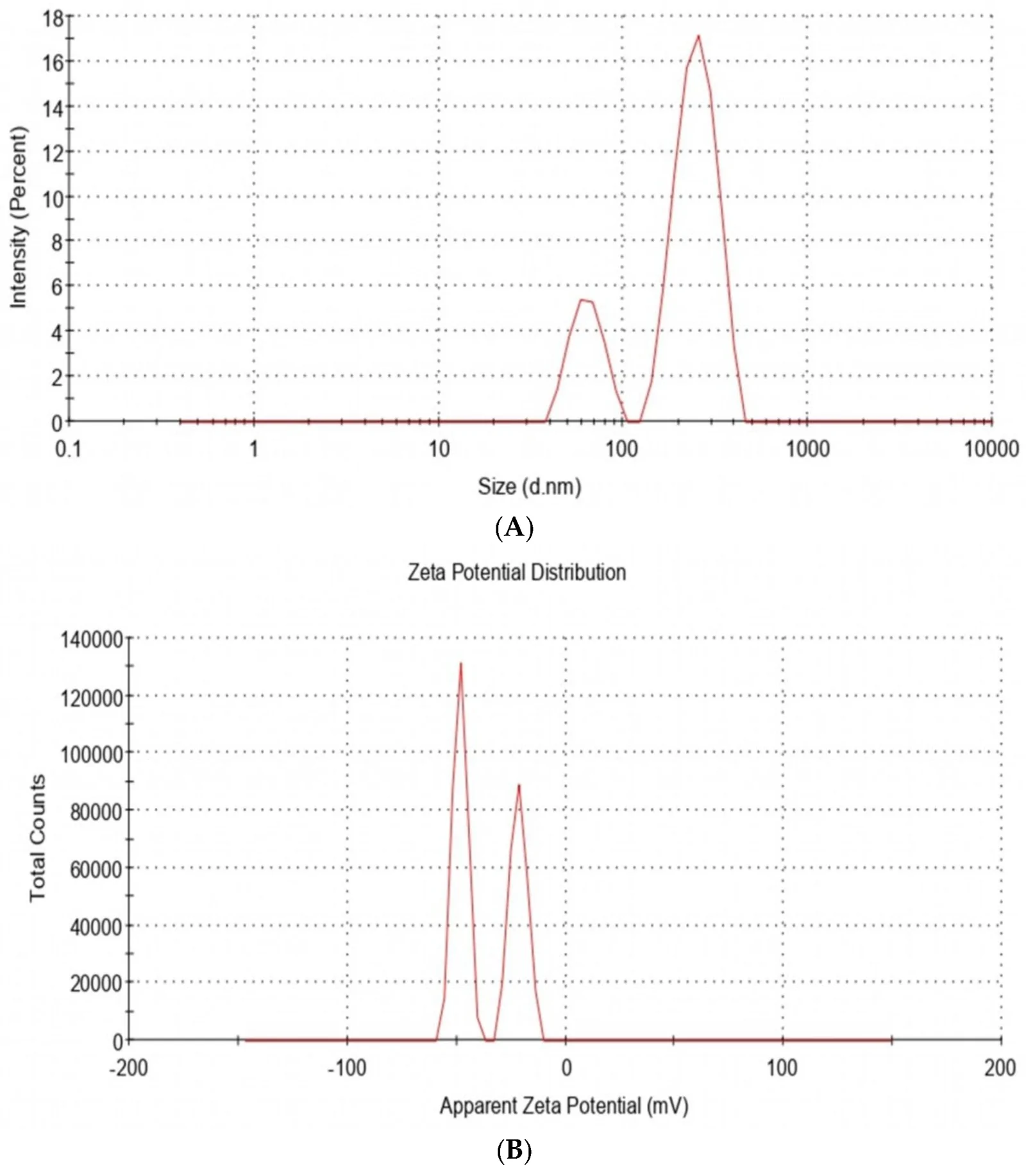
(**A**) The vesicle size distribution of the optimized lupeol-loaded bilosomes (LUPB). (**B**) The zeta potential of the optimized lupeol-loaded bilosomes (LUPB).

The particle size optimized formulation was found to be 300.9 nm (Figure 8A), where the distribution displays a major population at 253.0 nm (79.1% intensity) and a minor population at 64.21 nm (20.9% intensity). The secondary peak represents a smaller population (64.21 nm) rather than a larger, micron-sized particle (>1000 nm) population. This bimodal distribution may be consistent with the presence of smaller secondary vesicular structures and/or bile-salt-containing micellar species; however, DLS alone cannot conclusively distinguish these populations from other sources of size heterogeneity [28,45]. The PDI of optimized bilosome vesicles was found to be 0.328 ± 0.05 (n = 3) [46]. Similarly, the surface charge was found to be −37.7 mV where zeta potential distribution (Figure 8B) displays populations at −48.3 mV (55.7% area) and −21.6 mV (44.3% area). Both populations carry strong negative surface charges, with the dual peaks reflecting differential bile salt density and surface orientation between the primary bilosomes and the smaller co-existing micellar/vesicular structures.

The optimized LUPB formulation exhibited high entrapment efficiency (88 ± 1.14%), which is slightly higher than the reported values for bilosomal nanocarriers entrapping hydrophobic phytoconstituents. The high EE was obtained due to the lipophilic nature of lupeol [31,47,48]. The in vitro drug release percentage of the optimized LUPB formulation after 24 h was 84 ± 1.29%.

### 2.5. Characterization of Lupeol-Loaded Bilosomal Gel (LUPBG)

**Figure 9:**
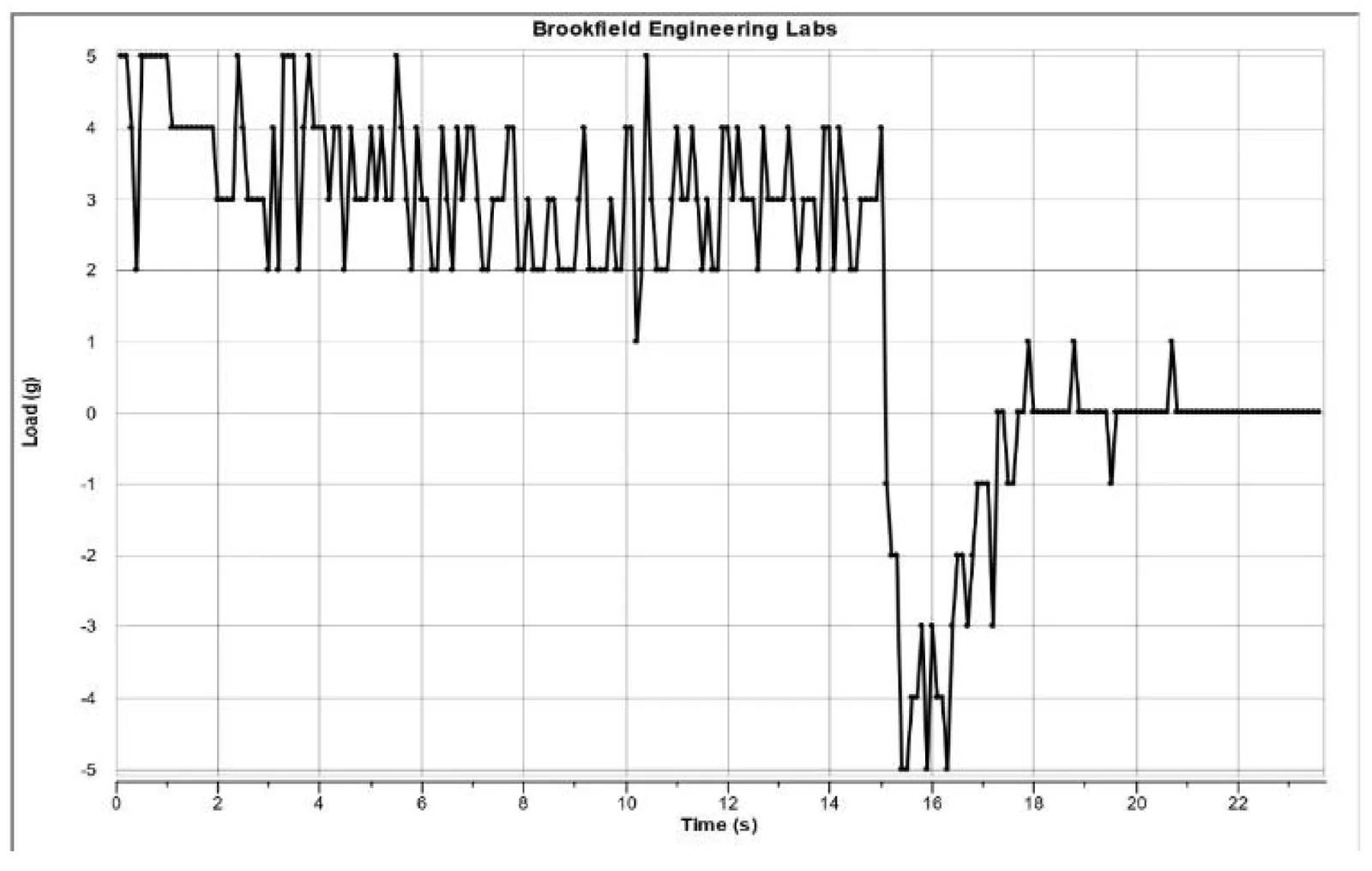
Texture analysis profile of the optimized lupeol-loaded bilosomal gel (LUPBG) showing adhesiveness, compressibility and cohesiveness.

The bilosomal gel was tested for its pH, spreadability, and drug content. The pH of the optimized bilosomal gel was found to be 6.08 ± 0.0402. The spreadability of the formulated gel was 4.2 ± 0.29 g.cm/s, and the drug content was as high as 96 ± 0.62%. The texture analysis graph (Figure 9) showed a maximum positive force of approximately 5 g. The presence of negative force during probe withdrawal (~−5 g) demonstrates good adhesiveness, suggesting strong interaction between the formulation and the probe surface.

The pH of the gel was in the range of the reported pH of psoriatic skin [49], which suggests good compatibility and therefore there would be no risk of skin irritation or minimal risk of irritation. A similar value of pH-topical formulation was reported by Montero et al. [50]. The obtained spreadability indicates adequate rheological characteristics that facilitate uniform application over the skin surface without drag. The demonstrated drug content reflects excellent drug loading and content uniformity.

### 2.6. In Vitro Drug Release and Release Kinetics of Lupeol-Loaded Bilosomal Gels

**Figure 10:**
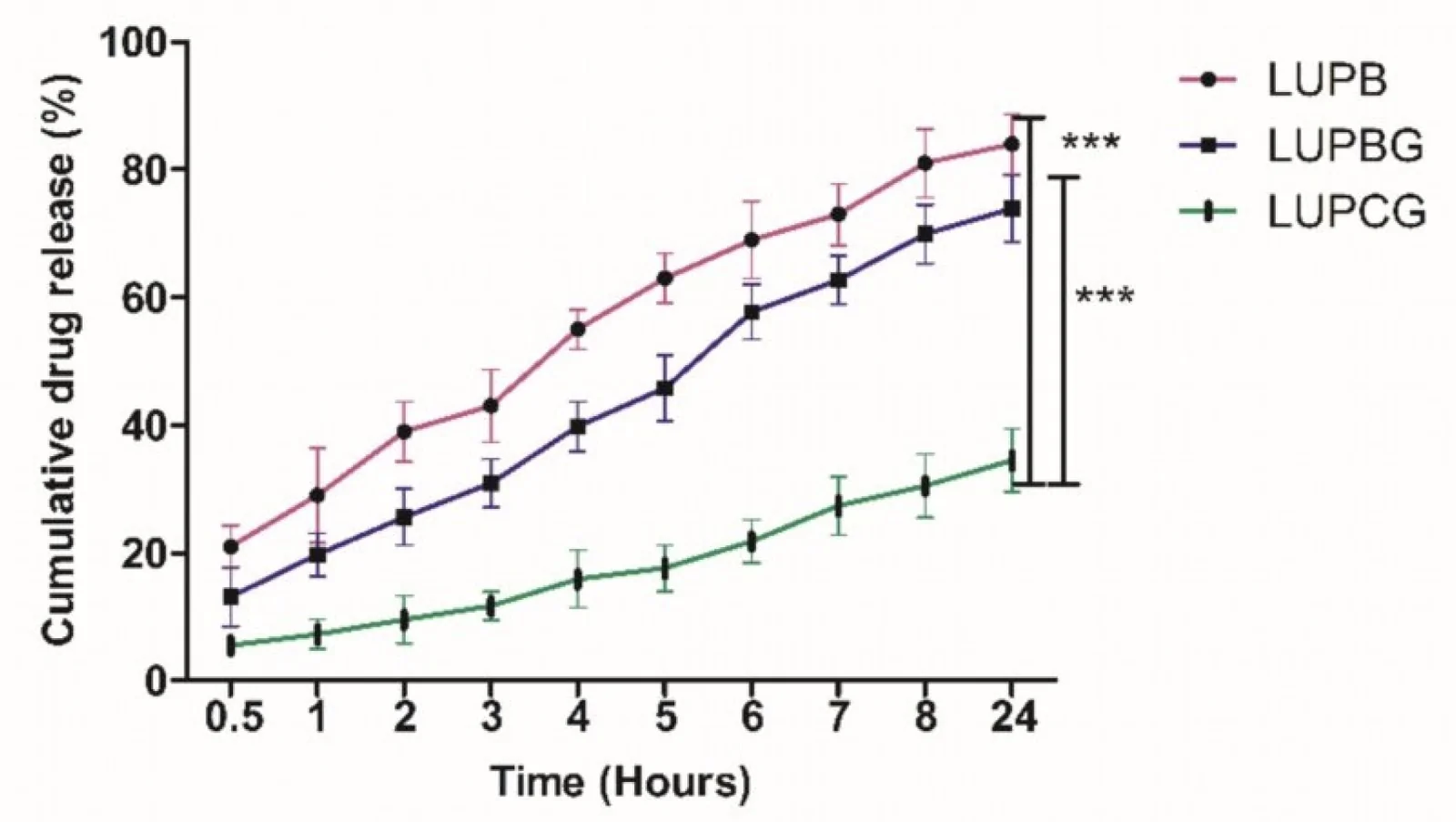
In vitro drug release profiles. Measurements were taken three times (n = 3) and statistical difference between the groups were calculated using two-way ANOVA followed by Bonferroni post-test. *** *p* < 0.001 vs. LUPCG. LUPB: lupeol-loaded bilosomes; LUPBG: lupeol-loaded bilosomal gel; LUPCG: lupeol-loaded conventional gel.

In vitro release of lupeol from lupeol bilosomal dispersion (LUPB), lupeol-loaded bilosomal gel (LUPBG), and lupeol-loaded conventional gel (LUPCG) formulations is demonstrated in Figure 10. After 24 h, LUPCG showed only 34.46 ± 2.43% drug release (*p* < 0.001), whereas LUPB dispersion exhibited a significantly higher release of 84 ± 1.29% (*p* < 0.001). The LUPBG formulation demonstrated 73.9 ± 1.21% drug release (*p* < 0.001), which was higher than LUPCG (34.46 ± 2.43%) and slightly lower than LUPB dispersion (84 ± 1.29%). The reduction in release is due to increased viscosity of the gel preparation. The bilosomal gel released more drug than the conventional lupeol gel, indicating that bilosomes improved drug solubilization.

Furthermore, the bilosomal vesicles exhibited a biphasic release profile, characterized by an initial burst release followed by sustained release over 24 h. Within the first hour, the initial drug release was 7.23 ± 2.30%, 19.7 ± 3.39% and 29 ± 5.42% from LUPCG, LUPBG, and LUPB dispersions, respectively. The initial burst phase is likely driven by quick dissolution of drug molecules adsorbed onto the vesicular surface. This biphasic release profile may provide a formulation-related advantage by combining an initial release phase with sustained drug release over 24 h; however, the clinical relevance of this profile requires further investigation [28]. Similar sustained-release behavior after incorporation of bilosomes into topical gels has been reported for berberine-, sulpiride-, clozapine- and simvastatin-loaded bilosomal gels [28,48,51,52,53]. The release kinetics of lupeol from the LUPBG was studied, and the data were fitted to zero-order, first-order, Higuchi, Hixson–Crowell, and Korsmeyer–Peppas models to understand the drug release mechanism. The Higuchi model provided the best fit (R^2^ = 0.9922), indicating that drug release was predominantly governed by diffusion through the thicker gel matrix (Table 7). The Rate Constant (K_H_ = 37.40) also indicates that the release of lupeol followed a diffusion-controlled mechanism [54]. This release behavior is consistent with kinetics observed in earlier investigations across various bilosomal formulations, such as acemetacin bilosomal gels, sildenafil citrate bilosomal gels, and lycopene bilosome dispersions [29,55,56].

### 2.7. Ex Vivo Permeation 

**Figure 11:**
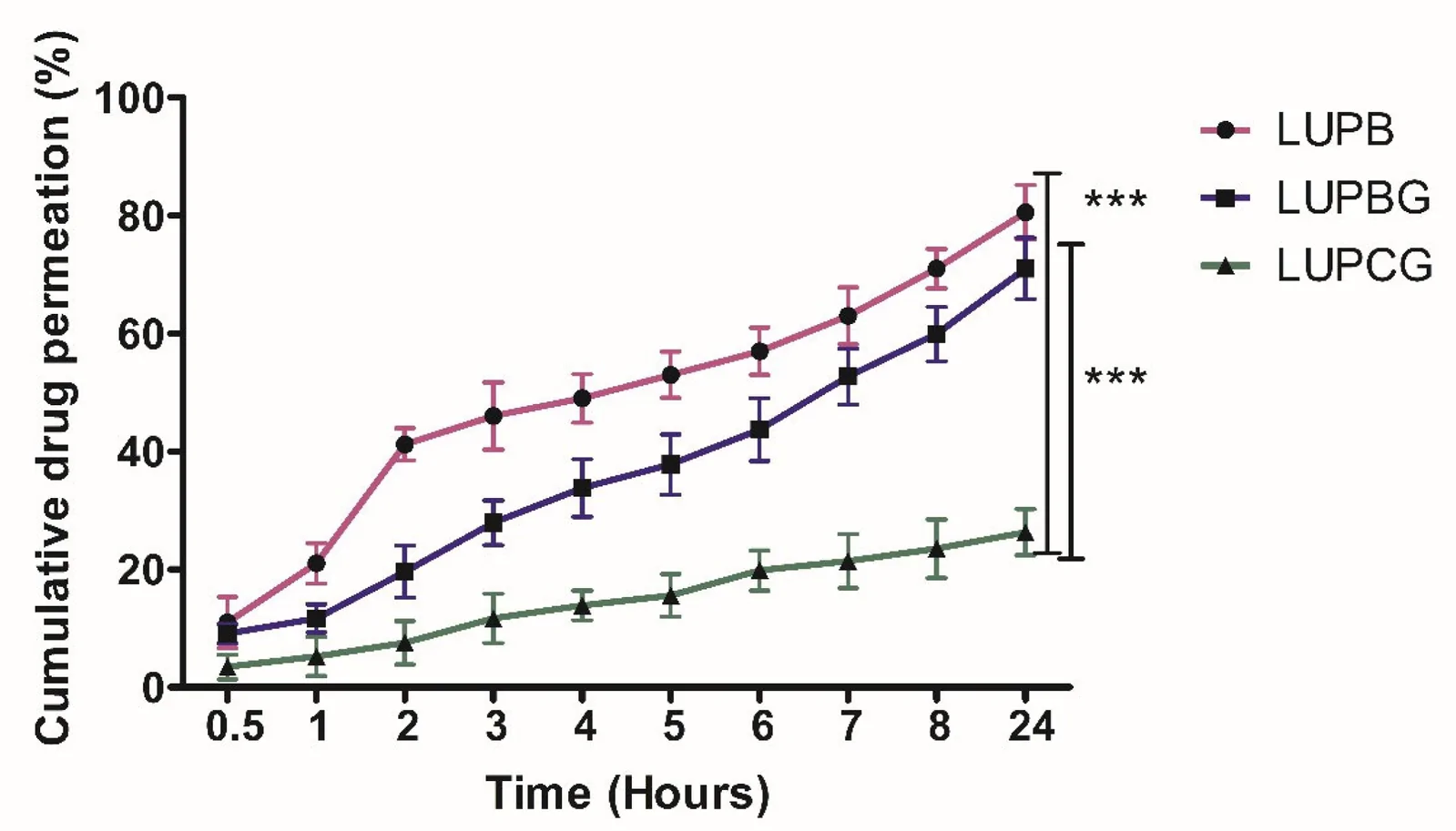
Ex vivo skin permeation profiles of lupeol. Measurements were taken three times (n = 3), and statistical differences between the groups were calculated using two-way ANOVA followed by Bonferroni post-test. *** *p* < 0.001 vs. LUPCG. LUPB: lupeol-loaded bilosomes; LUPBG: lupeol-loaded bilosomal gel; LUPCG: lupeol-loaded conventional gel.

The ex vivo skin permeation study showed that the bilosomal gel (LUPBG) significantly enhanced lupeol permeation across the skin compared with the conventional gel (LUPCG), as shown in Figure 11. The maximum drug permeation was observed with LUPB (80.53 ± 0.64%, n = 3, *p* < 0.001). LUPBG exhibited 71 ± 1.21% drug permeation (n = 3, *p* < 0.001), which was significantly higher than that of LUPCG (26.31 ± 0.93%, *p* < 0.001). The enhanced drug permeation may be attributed to the presence of sodium deoxycholate, which may increase vesicle deformability and facilitate penetration through the stratum corneum [24,57]. Sodium deoxycholate in bilosomes acts as an edge activator, potentially destabilizing the lipid bilayers and increasing membrane flexibility. This mechanism may increase vesicle deformability, thereby facilitating their passage through the skin and enhancing drug delivery. Al-Mahallawi et al. reported a similar ex vivo permeation study using tenoxicam-loaded bilosomes [58]. The literature suggests that bile salts enhance skin permeation by fluidizing stratum corneum lipids and increasing vesicle flexibility, allowing bilosomes to interact with and potentially traverse the skin via transcellular (through corneocytes), intercellular (between corneocytes), and appendageal pathways (via sweat glands and hair follicles), thereby potentially facilitating deeper drug delivery [36,59,60,61]. In addition, bile salts can interact with and bind calcium ions at cell junctions. This calcium depletion may temporarily weaken cell-to-cell adhesion and loosen tight junctions, thereby enhancing the paracellular transport of encapsulated drugs [62].

Moreover, optimized bilosomal gel LUPBG demonstrated substantially higher lupeol flux after 24 h (Jss_24h_ = 91.41 µg/cm^2^/h) than the conventional gel LUPCG (Jss_24h_ = 33.71 µg/cm^2^/h), with a 2.71-fold enhancement in flux. Lupeol bilosomal dispersion (LUPB) showed a higher flux (103.24 µg/cm^2^/h) than LUPBG (91.41 µg/cm^2^/h); however, the difference was not significant, with less than a two-fold difference (1.129-fold enhancement). Thus, the optimized formulation successfully improves the transdermal permeation of lupeol. The permeability coefficient (Kp) after 24 h was found to be 0.103 for LUPB, 0.0914 cm/h for the LUPBG and 0.0359 cm/h for the LUPCG.

### 2.8. In Vivo Study Result

Psoriasis is a chronic immune-mediated inflammatory disorder characterized by excessive keratinocyte proliferation and angiogenesis into the epidermis and dermis. The inflammatory cytokines such as TNF-α play a crucial role in disease progression [63,64].

Imiquimod (IMQ) is a chemical compound that induces plaque-type psoriasis-like chronic inflammation and was used to induce human plaque-type psoriasis. IMQ induces dermatopathy when applied topically to mice, characterized by elevation in inflammatory cytokines, erythema, scaling, and epidermal hyperproliferation [65]. In the present study, topical application of IMQ successfully induced severe erythema, scaling and skin thickening in epidermal skin.

#### 2.8.1. Psoriatic Area Severity Index (PASI) Score

Psoriatic Area Severity Index (PASI) score reflects changes in key clinical features, including redness (erythema), thickness (induration), and scaling (desquamation), providing a quantitative measure of treatment-related improvement in the imiquimod-induced psoriasis-like animal model. Aforementioned core clinical features of psoriasis (Erythema, induration, and desquamation) were monitored throughout the study.

**Figure 12:**
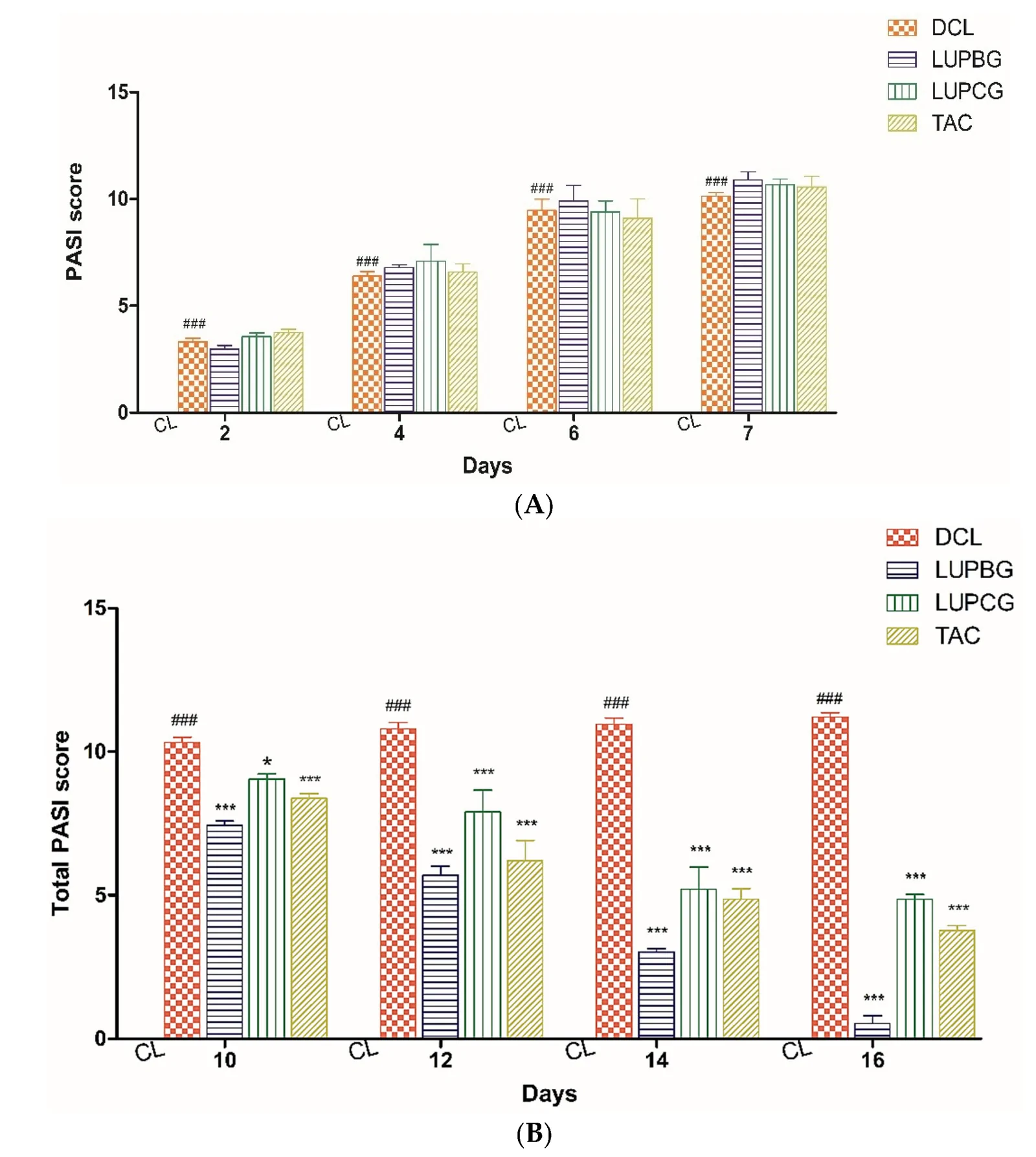
(**A**) PASI score of animals during disease induction phase. Statistical differences between the groups were calculated using two-way ANOVA followed by Bonferroni post-test. ### *p* < 0.001 vs. CL. (**B**) PASI score of animals during disease treatment phase. Statistical differences between the groups were calculated using two-way ANOVA followed by Bonferroni post-test. ### *p* < 0.001 vs. CL, *** *p* < 0.001, * *p* < 0.05. CL: healthy control, DCL: diseased control, TAC: tacrolimus standard, LUPBG: lupeol-loaded bilosomal gel, LUPCG: lupeol-loaded conventional gel.

During the disease induction phase (1–7 days), topical application of 5% imiquimod (IMQ) cream gradually increased PASI scores in all groups except the CL group, which confirms the successful establishment of the psoriasis mouse model in Figure 12A.

In the treatment phase (8–16 days), the PASI score gradually reduced in the groups treated with LUPBG, LUPCG and standard drug tacrolimus (TAC). Maximum reduction in PASI score was observed on day 16. The average PASI score for the disease control group (DCL) was 11.21 ± 0.15, the TAC group showed obvious improvements (PASI 3.77 ± 0.11, *p* < 0.001), whereas the LUPBG group showed a significantly lower score of 0.53 ± 0.27 (*p* < 0.001), indicating marked improvement in psoriasis-like inflammation on day 16. These improvements revealed the effectiveness of lupeol-loaded bilosomal gel (LUPBG) in normalizing the psoriatic alterations induced by IMQ, as depicted in Figure 12B. Similar improvements in PASI scores have been reported with other lipid-based nanocarrier systems. Rashid SA et al. evaluated methotrexate-loaded lipid nanocarriers for treating IMQ-induced psoriasis-like inflammation [66], while Sawant et al. developed curcumin-loaded bilosomes for psoriasis management [67], both demonstrating improved PASI scores. The LUPCG group of lupeol-loaded conventional gel still showed sustained symptoms (PASI 4.86 ± 0.17, *p* < 0.001) to those in the disease control animals.

#### 2.8.2. Determination of Spleen Index

Spleen enlargement reflects systemic immune activation during inflammation, and the spleen index (relative spleen weight) can be used as an indicator of immune stimulation and inflammatory severity in IMQ-induced psoriasis-like mice. Shinno-Hashimoto et al. highlighted the important role of spleen in chronic inflammation in IMQ-treated mice, reporting significant increases in splenic neutrophils, proerythroblasts, B cells, macrophages, and dendritic cells, accompanied by increased spleen weight [68]. In a recent study, Sawant and Mallya (2026) evaluated spleen weight as an indicator of systemic inflammatory response and reported improved spleen-related outcomes following treatment with quercetin-loaded bilosomal gel in IMQ-induced psoriasis-like mice [67]. In this study, the spleen index was evaluated in the control group (CL), disease control group (DCL), tacrolimus-treated group (TAC), along with LUPBG and LUPCG groups to assess the systemic inflammatory response associated with psoriasis progression. The DCL group exhibited a markedly elevated spleen index (1.03 ± 0.06, *p* < 0.001) compared with the CL group (0.43 ± 0.02, *p* < 0.001). The increased spleen index observed in the DCL group reflects excessive immune cell proliferation and inflammatory activation.

**Figure 13:**
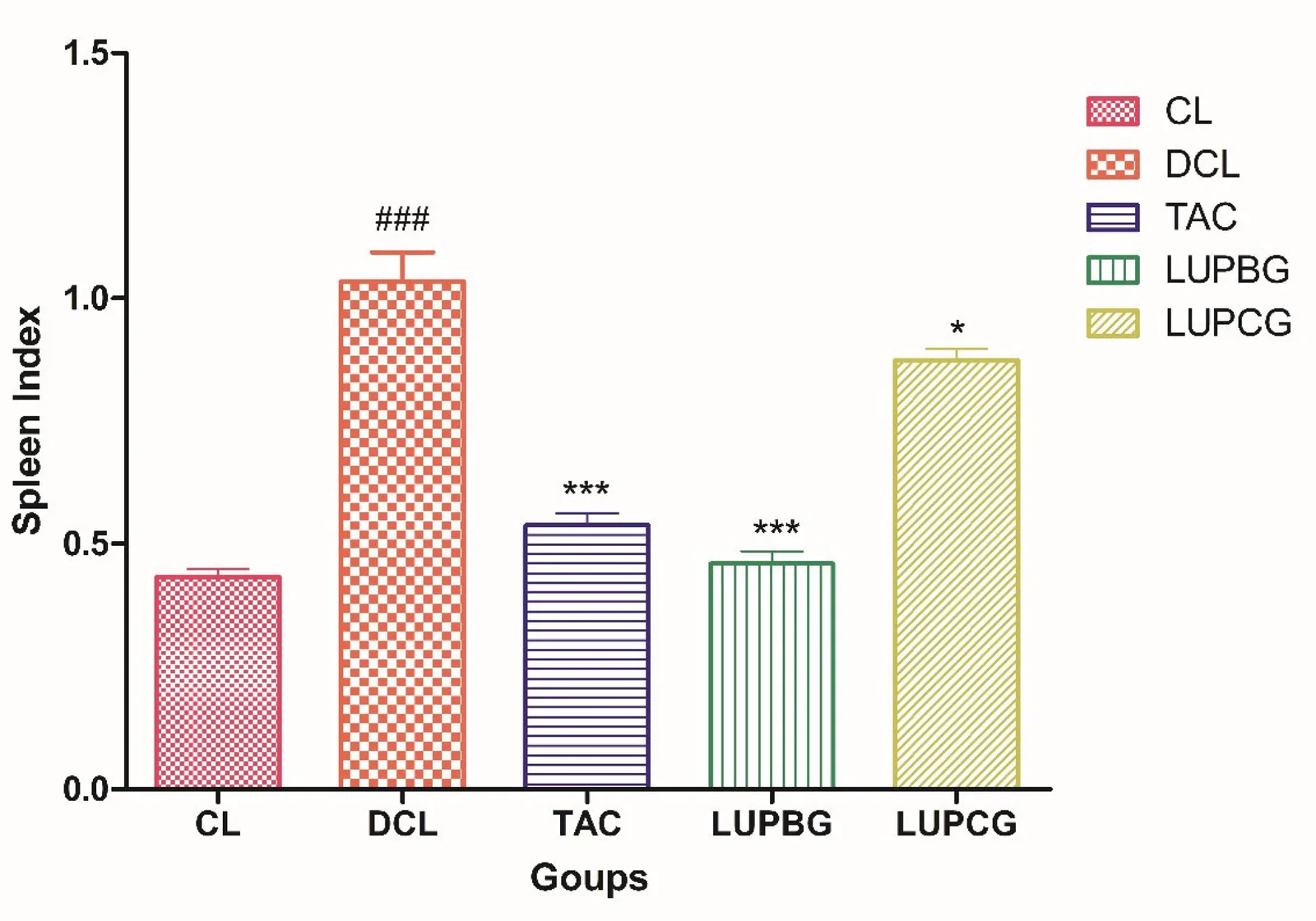
Spleen index of mice. Data are presented as Mean ± SEM, n = 6. Statistical difference between the groups were calculated using one-way ANOVA followed by Tukey’s multiple comparison test. ### *p* < 0.001 vs. DCL, *** *p* < 0.001 vs. DCL, * *p* < 0.05 vs. DCL. CL: healthy control, DCL: diseased control, TAC: tacrolimus standard, LUPBG: lupeol-loaded bilosomal gel, LUPCG: lupeol-loaded conventional gel.

Treatment with the TAC group significantly reduced the spleen index to 0.50 ± 0.03 (*p* < 0.001), demonstrating a reduction in systemic inflammation, which suggests strong anti-inflammatory activity of the developed formulation. Similarly, the LUPBG group showed a reduction in spleen index (0.45 ± 0.02, *p* < 0.001), while LUPCG showed only a partial reduction in spleen index (0.87 ± 0.04, *p* < 0.001) compared with the DCL group (Figure 13). Other studies also reported that nano formulations decrease spleen weight and inflammatory mediators in experimental psoriasis [68,69].

#### 2.8.3. Effects of Interventions on Blood Parameters

**Figure 14:**
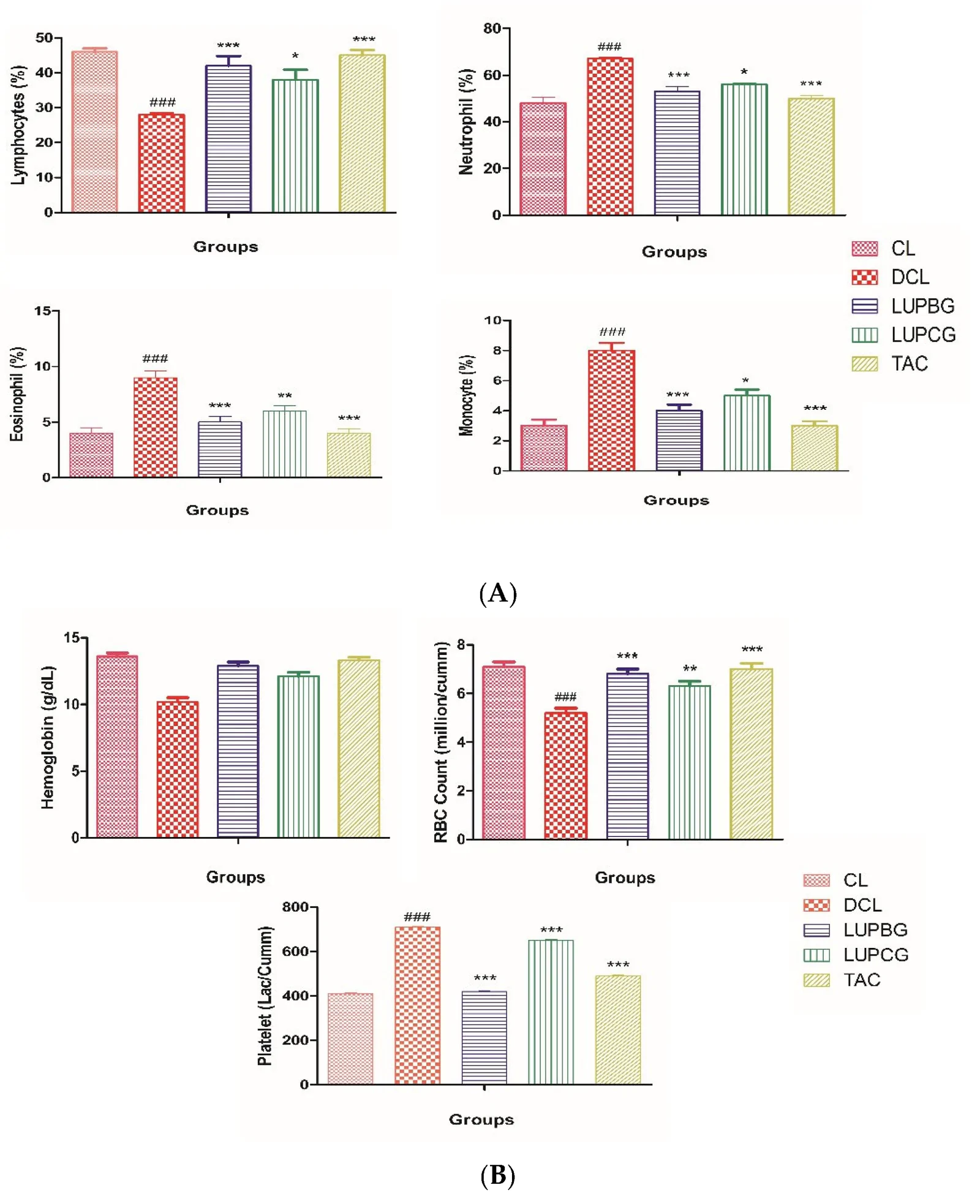
(**A**) Evaluated blood parameters (lymphocytes, neutrophil, eosinophil, and monocyte) of mice on day 16. Statistical differences between the groups were calculated using One-way ANOVA followed by Tukey’s multi comparison test. ### *p* < 0.001 vs. CL, *** *p* < 0.001 vs. DCL, ** *p* < 0.01 vs. DCL, * *p* < 0.01 vs. DCL. (**B**) Evaluated blood parameters (hemoglobin, RBC count and platelet) of mice on day 16. Statistical difference between the groups were calculated using one-way ANOVA followed by Tukey’s multi comparison test. ### *p* < 0.001 vs. CL, *** *p* < 0.001 vs. DCL, ** *p* < 0.01 vs. DCL. CL: healthy control, DCL: diseased control, TAC: tacrolimus standard, LUPBG: lupeol-loaded bilosomal gel, LUPCG: lupeol-loaded conventional gel.

Inflammation in psoriasis is also associated with blood parameters, because inflammatory cytokines influence hematopoiesis. The anti-inflammatory activity of lupeol was reported by Geetha et al. [70]. In the present study, blood parameters were successfully restored after treatment with lupeol-loaded bilosomal gel. Similar restoration of blood parameters has been reported in the psoriatic mouse model [68]. Evaluation of changes in hematological parameters was carried out using the complete blood count (CBC) test, and the results are presented in Table 8. The data show the level of significance among the CL, DCL, LUPBG, LUPCG, and TAC groups (Figure 14A,B).

A significant reduction in hemoglobin concentration was observed in the DCL group (10.2 ± 0.34 g/dL, *p* < 0.001) compared with CL group animals (13.6 ± 0.25 g/dL). Treatment with bilosomal gel restored hemoglobin levels toward normal. The LUPBG group (12.9 ± 0.27 g/dL, *p* < 0.001) showed greater improvement than LUPCG group (12.1 ± 0.3 g/dL, *p* < 0.01), while the TAC group exhibited a hemoglobin level of 13.3 ± 0.24 g/dL (*p* < 0.001). The RBC count decreased from 7.1 ± 0.20 million/cumm in CL group to 5.2 ± 0.16 (*p* < 0.001) million/cumm in the diseased group. LUPBG restored the RBC count to 6.8 ± 0.19 (*p* < 0.001) million/cumm, whereas LUPCG achieved a value of 6.3 ± 0.18 (*p* < 0.01) million/cumm. Platelet count was also increased in DCL group (710 ± 1.3 lac/cumm, *p* < 0.001) compared with CL group (427 ± 2 lac/cumm, *p* < 0.001). LUPBG and LUPCG group significantly improved platelet count 421 ± 2.1 lac/cumm and 650 ± 2.5 lac/cumm (*p* < 0.001) respectively. Lee et al. previously investigated the association between hemoglobin levels and psoriasis risk in patients with chronic kidney disease using Korean National Health Insurance data. Their findings showed that lower hemoglobin levels were associated with increased psoriasis risk, although causality remains unclear, suggesting that low hemoglobin may serve as a marker of psoriasis susceptibility [71]. Supporting this connection, Helmi et al. recently reported that hemoglobin concentrations drop progressively with rising disease activity, decreasing from 12.80 ± 1.95 g/dL in moderate disease to 11.30 ± 1.60 g/dL in highly active disease [72].

The different leukocyte count demonstrated an inflammatory response in the diseased animals. Neutrophil increased from 48 ± 2% in CL group to 67.0 ± 1.62% (*p* < 0.001) whereas lymphocyte reduced from 46 ± 2% in CL group to 28.0 ± 1.79% (*p* < 0.001) in DCL group. In treatment group, neutrophil and lymphocytes levels were reduced to 53.0 ± 1.3% (*p* < 0.001) and 42.0 ± 1.8% (*p* < 0.001) in LUPBG group, while a moderate improvement (56.0 ± 1.7% neutrophils and 38.0 ± 2.1% lymphocyte) was observed in LUPCG group. Similarly, eosinophil and monocyte counts were markedly elevated (9.0 ± 0.7% eosinophil and 8.0 ± 0.27% monocytes) in the DCL group compared to the CL group. Both treatments reduced eosinophil and monocyte percentages.

The overall results indicated that those values of CBC derived from LUPBG were closer to those values retrieved from the CL group, which also indicated that the prepared bilosomal gel can cure psoriasis in the IMQ-induced mice model [73]. Liu et al. also treated the psoriatic mice with glycyrol (a natural compound from licorice root) and reported a similar type result of blood parameters [74]. Consistent with our findings, Zhou G. et al. suggested that eosinophil count, neutrophil-to-lymphocyte ratio (NLR), and lymphocyte-to-monocyte ratio (LMR) may serve as potential indicators for evaluating treatment response, given their association with psoriasis severity [75]. In the present study, the calculated NLR values showed a similar pattern, with markedly higher values in diseased control (DCL) group (NLR: 2.39) compared with the healthy control group (CL) group (NLR: 1.04). Treatment with LUPBG reduced these values to 1.26 for NLR, bringing them closer to the healthy control group (CL) values. Thus, LUPBG treatment effectively reduces systemic inflammation by successfully restoring these inflammatory markers back toward normal, healthy control levels.

Similar findings were reported by Wang W-M et al. where patients with psoriasis had higher WBC, neutrophil, and platelet counts, as well as increased NLR, but lower hemoglobin, lymphocyte, and RBC counts compared with healthy controls [76]. Şener G. et al. also reported that increased WBC, neutrophil, monocyte, NLR, and monocyte–lymphocyte ratio (MLR) levels correlated with psoriasis severity and decreased following treatment, supporting their use alongside PASI for monitoring disease severity and treatment response [77]. In another study, Zhang Y et al. suggested that neutrophil-to-lymphocyte ratio (NLR), monocyte-to-lymphocyte ratio (MLR) may serve as useful biomarkers for evaluating systemic inflammation and disease severity in patients with psoriasis.

#### 2.8.4. Estimation of Tumour Necrosis Factor-Alpha (TNF-α)

**Figure 15:**
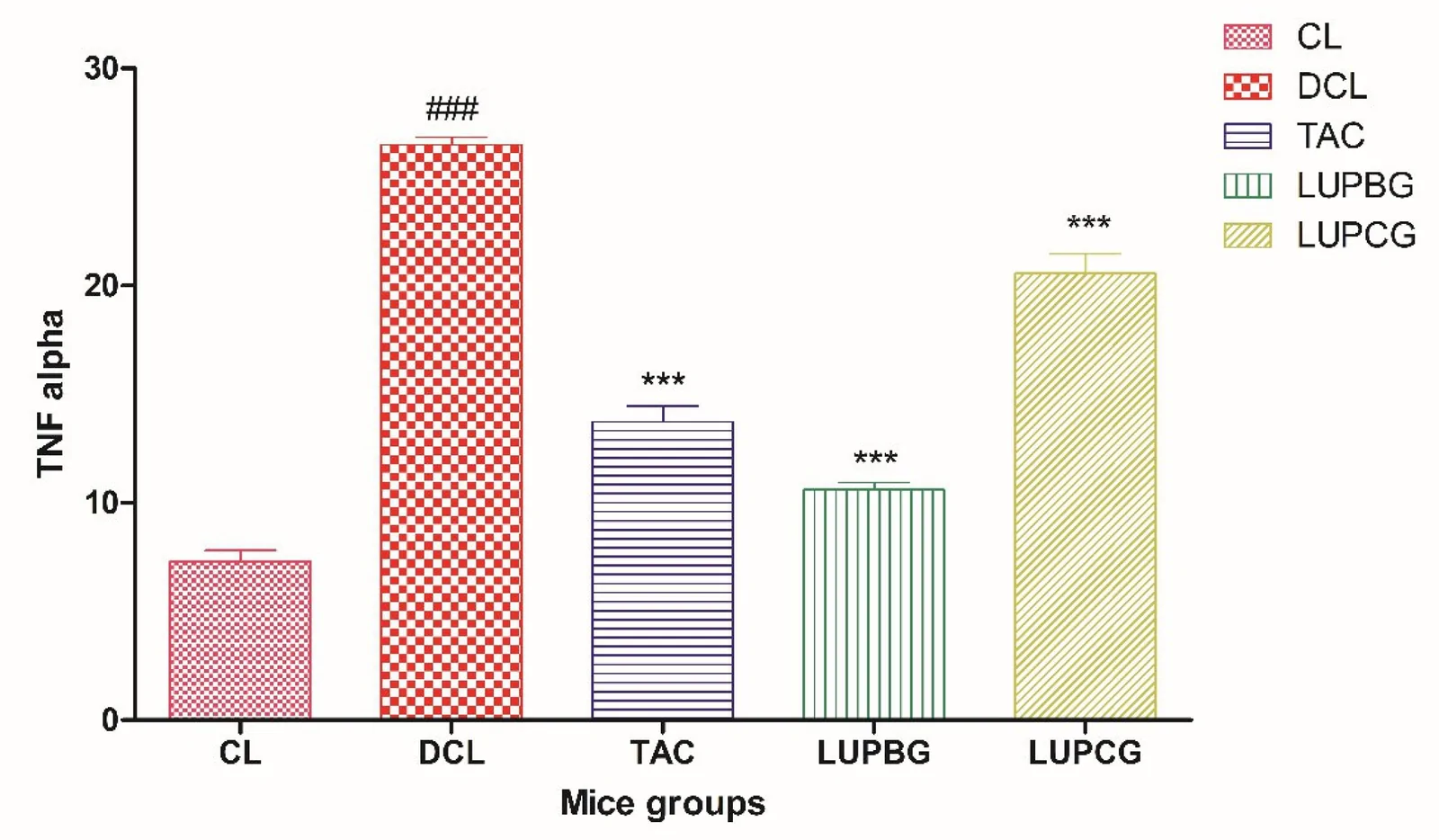
TNF-α of different groups of mice on day 16. Data is presented as Mean ± SEM, n = 6. Statistical differences between the groups were calculated using one-way ANOVA followed by Tukey’s multiple comparison test. ### *p* < 0.001 vs. CL, *** *p* < 0.001 vs. DCL. CL: Healthy control, DCL: Diseased control, TAC: tacrolimus standard, LUPBG: lupeol-loaded bilosomal gel, LUPCG: lupeol-loaded conventional gel.

Psoriasis stems from stressed keratinocytes releasing pro-inflammatory cytokines like IL-6, IL-1β, and TNF-α, triggering interferon-α (IFN-α) production by dendritic cells. Subsequent activation of lymph node dendritic cells drives epidermal hyperproliferation and skin inflammation [78]. In the current study, disease control group (DCL) exhibited significantly elevated skin TNF-α levels (26.00 ± 0.45 pg/mL, *p* < 0.001) compared with the healthy control group (CL), confirming enhanced inflammatory activity (Figure 15). In a similar study, Abdelrahman et al. reported a significant increase in TNF-α immunoreactivity in mice with imiquimod-induced psoriasis-like skin inflammation. TNF-α expression was significantly reduced in treatment groups (TAC, LUPBG, and LUPCG). Levels in the TAC group (Tacrolimus cream) were 13.74 ± 0.59 pg/mL (*p* < 0.001), which further decreased to 10.61 ± 0.91 pg/mL (*p* < 0.001) in the LUPBG group [79]. In a parallel study, Zhang et al. reported inhibition of inflammatory cytokines, including TNF-α, in the skin of IMQ-induced psoriatic mice, with inhibition increasing at higher doses [80]. The lowest downregulation of TNF-α was observed in the LUPBG group in comparison to the TAC and LUPCG groups. These findings marked the more potent anti-inflammatory activity of the lupeol-loaded bilosomal gel formulation, which translated into the improvements recorded in the PASI scores and the spleen index, and hence the superior therapeutic potential to conventional treatments. The reduction in TNF-α levels observed in the LUPBG group is consistent with previous reports demonstrating that lupeol suppresses inflammatory cytokine production and modulates immune responses [70,81].

#### 2.8.5. Effects of Interventions on Histopathology

**Figure 16:**
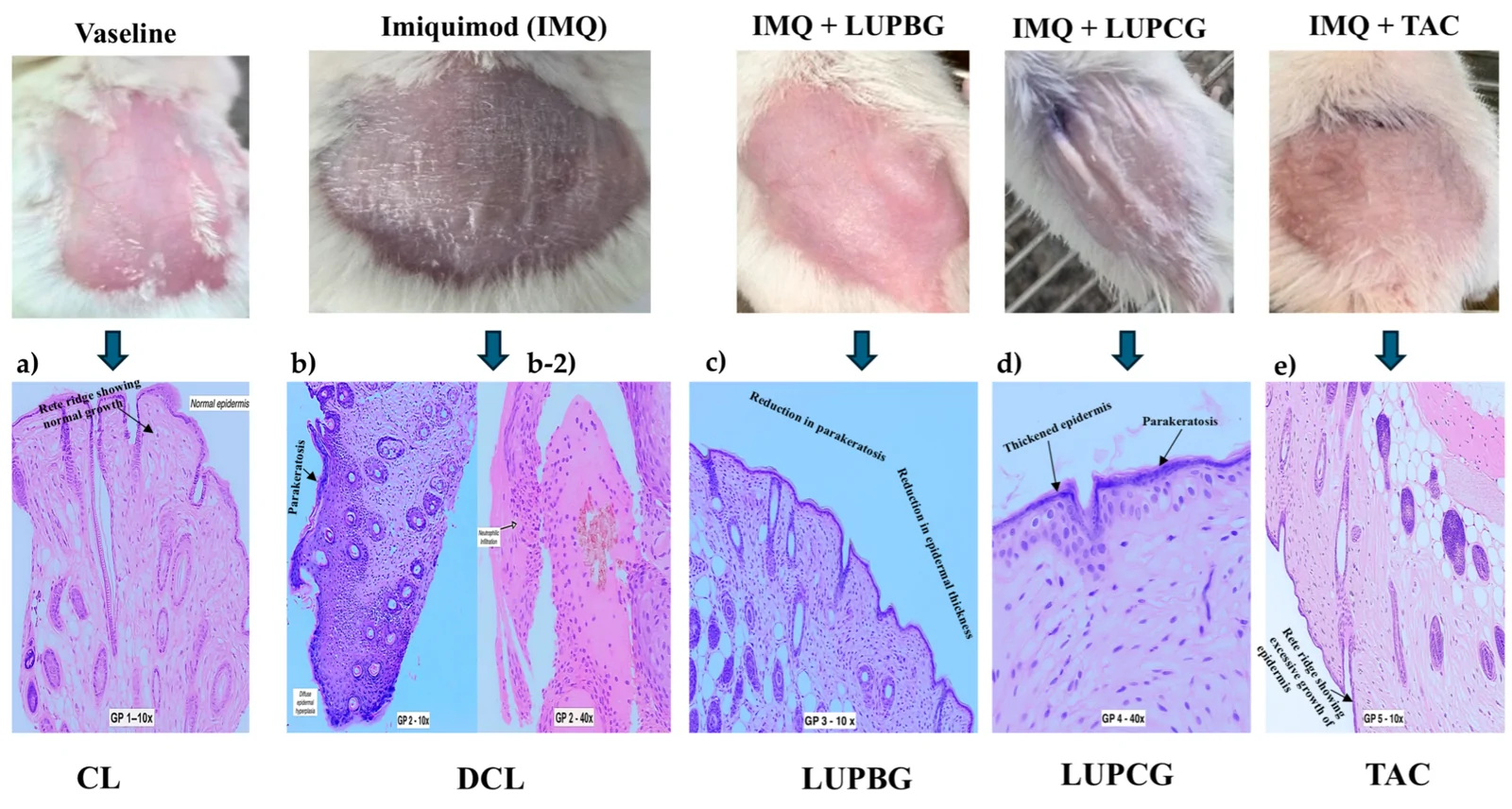
Representative gross morphological and histopathological images of dorsal skin from mice in the (a) healthy control (CL), (b) disease control (DCL) at 10X, (b-2) disease control (DCL) at 40X (c) lupeol-loaded bilosomal gel (LUPBG), (d) lupeol-loaded conventional gel (LUPCG), and (e) tacrolimus-treated (TAC) groups on day 16 following IMQ-induced psoriasis. Gross images show the macroscopic appearance of the dorsal skin, while H&E-stained sections illustrate the corresponding histopathological changes in each group.

The effect of standard drug (TAC), bilosomal gel (LUPBG), and conventional gel (LUPCG) on psoriatic-like lesions was evaluated by histopathology in the imiquimod (IMQ)-induced mice model. Representative histopathological images of all experimental groups are presented in Figure 16. The dorsal skin from the CL group showed normal epidermal and dermal architecture without any signs of inflammation [82]. In contrast, the DCL group exhibited marked epidermal hyperplasia (acanthosis), parakeratosis, extensive inflammatory cell infiltration in the dermis, which are histologic features of psoriasis. Application of 5% imiquimod cream induced similar psoriatic histopathological alterations in the murine epidermal and dermal layers [83].

Treatment with the standard cream (tacrolimus) attenuated the inflammatory signs more moderately, as evidenced by partial restoration of skin layers and reduced cell infiltration. TAC group showed partial restoration of normal skin architecture with reduced epidermal thickness and decreased inflammatory cell infiltration.

Notably, the LUPBG group demonstrated marked histological alleviation with low epidermal thickening, minimum inflammatory cell infiltration, and restoration of normal skin architecture, indicating successful normalization of psoriatic symptoms [83].

In comparison, the LUPCG-treated group exhibited moderate improvement in thickness of the epidermis. The LUPCG group showed a sustained massive thickening of the epidermis and moderate inflammation, which indicated scarce therapeutic efficiency. These data indicate that bilosomes play an essential role in the recovery of skin architecture in psoriasis at the histological level, showing better results in comparison with those in conventional formulations. Similar improvements in imiquimod-induced psoriatic histopathological changes were reported by Sawant and Mallya (2026) following topical application of quercetin-loaded bilosomes [67]. Fathalla et al. (2020) similarly reported improved psoriatic plaque histopathology following treatment with anthralin-loaded ethosomal and liposomal gels [84].

Overall, the present study demonstrates that the lupeol-loaded bilosomal gel effectively treated IMQ-induced psoriasis with significant improvements in PASI score, spleen index, TNF-α level, hematological parameters, and histopathological assessments. It indicates that bilosomal delivery enhances the therapeutic performance of lupeol-loaded bilosomal gel (LUPBG) compared with conventional gel (LUPCG).

## 3. Conclusions

This study successfully developed and optimized a lupeol-loaded bilosomal gel as an advanced topical delivery system for psoriasis using a Box–Behnken design. The optimized formulation exhibited nanosized vesicles, high entrapment efficiency, enhanced skin permeation, and marked anti-psoriatic effects compared with the conventional lupeol gel and standard tacrolimus cream. The improved formulation performance supports the potential of bilosomes to enhance the topical delivery of poorly water-soluble lupeol and may contribute to improved localized treatment of psoriasis.

Despite the promising anti-psoriatic efficacy of the lupeol-loaded bilosomal gel, certain study limitations warrant consideration. First, the IMQ-induced psoriasis model reproduces key features of human plaque psoriasis, but it does not fully reflect the complex, chronic, and relapsing immune-mediated pathophysiology of human psoriasis. Furthermore, dermal toxicity, systemic exposure, pharmacokinetic behavior, and long-term formulation stability were not evaluated in the present study.

Future studies should evaluate the optimized lupeol-loaded bilosomal gel in additional clinically relevant animal models, followed by well-designed clinical trials to confirm safety, tolerability, efficacy, and patient acceptability. Pharmacokinetic and skin-distribution studies should assess local drug retention along with systemic exposure. Long-term stability, scalability, and formulation reproducibility should also be investigated before clinical translation. Mechanistic studies, including comprehensive molecular and gene-expression profiling, are needed to further elucidate the molecular signaling pathways potentially involved in the effects of lupeol-loaded bilosomes and to further support their therapeutic potential for long-term psoriasis management. Overall, this work highlights the potential of bilosomes as an effective nanocarrier platform for topical delivery of lupeol and provides a potential strategy for further investigation in the topical management of psoriasis.

## 4. Materials and Methods

### 4.1. Materials

Lupeol (LP) was procured as a gift sample from Sami-Sabinsa group limited, Bangalore, India. HPLC-grade acetonitrile, water, and formic acid were procured from Merk Life Science Pvt. Ltd., Mumbai, India. Sodium deoxycholate (SDC) was obtained from Central Drug House, New Delhi, India. Carbopol^®^ 934P, Methyl paraben, Propyl paraben, Triethanolamine (TEA), Sorbitan Mono Stearate (Span 60^®^) and Tween 20, Cholesterol, Formaldehyde, Hematoxylin and Eosin were sourced from Himedia, Mumbai, India. Chloroform was obtained from CDH^®^, New Delhi, India. The dialysis membrane (molecular weight cut-off of 12–14 kDa) was purchased from HiMedia Laboratories Pvt. Ltd. (Mumbai, India). Imiquimod cream (5% *w*/*w*; Glenmark Pharmaceuticals, Local market) was procured commercially from the market. The enzyme-linked immunosorbent assay (ELISA) kit (TNF-α) was procured from Laczine Biosciences, Jaipur, Rajasthan, India.

### 4.2. Methods

#### 4.2.1. High-Performance Liquid Chromatography (HPLC)

The Ultra High-Performance Liquid Chromatograph (UHPLC) of Nexera series (Shimadzu Corporation, Kyoto, Japan) with quaternary solvent delivery pump, auto sampler, and photodiode array detector (PDA) was used for the chromatographic analysis of lupeol. UHPLC using a reverse phase column (, Accucore column C18, 2.6 μm, 2.1 × 150 mm, Thermo Scientific, Waltham, MA, USA) was used to optimize the chromatographic separation. The mobile phase contained a mixture of 0.1% formic acid in water (A) and 0.1% formic acid in acetonitrile (B) with a gradient elution system which was started with 10% of solvent B (0–5.0 min), followed by transitioning up to 30% of solvent B (10–15 min), Afterward this eluent system was increased to 50% of solvent B (15.0–20.5 min) which reached up to 90% of solvent B (20.5–25.0 min), and finally decreasing from 90% to 10% of solvent B for 25–30 min time period. The flow rate, injection volume and column temperature were maintained at 0.5 mL/min, 5.0 μL and 40 °C, respectively. Detection was carried out at 210 nm wavelength.

#### 4.2.2. Drug–Excipient Interaction Study (FTIR)

To determine the compatibility of lupeol with the excipients, the FTIR spectra of lupeol, cholesterol, and the lyophilized optimized formulation were obtained using a Nicolet Summit X FTIR spectrometer (Thermo Fisher Scientific, Madison, WI, USA) equipped with an Everest ATR accessory in the 4000–450 cm^−1^ range. One sample scan and one background scan were recorded using a DTGS KBr detector and KBr beamsplitter. The spectra were analyzed for changes in characteristic peak positions and the appearance or disappearance of absorption bands to evaluate possible drug–excipient interactions [85].

#### 4.2.3. Preparation of Lupeol-Loaded Bilosomes (LUPB)

Lupeol-loaded bilosomes (LUPB) were prepared using the modified thin-film hydration method [33,86]. Lupeol (10 mg), Span 60 (50–70%), and cholesterol (10–30 mg) were accurately weighed and dissolved in 10 mL of chloroform. This mixture was subjected to bath sonicator for 10–15 min to ensure complete dissolution. This organic solution was transferred into a round-bottomed flask (RBF) and rotary evaporated at 50 °C for 45 min at 120 RPM to form a thin, dry lipid film in the inner wall of the flask. This dry film was then rehydrated with 10 mL of bile salt (sodium deoxycholate, 10 mg) containing phosphate buffer (pH 6.8) for 30 min to obtain a dispersion of lupeol-loaded bilosomes. The nano-dispersion was passed through a 0.45 μm syringe filter as a sample-processing step before further characterization. The resulting opalescent dispersion of bilosomes was sonicated at specified time at 20–25 kHz using a probe sonicator (PRO650, Labman Probe Sonicator, Chennai,India) and stored at 4 °C until further use.

#### 4.2.4. Optimization Procedure of Lupeol-Loaded Bilosomes (LUPB)

The optimization of prepared LUPB was carried out using Box–Behnken Design (BBD) in Design-Expert^®^ version 13.0.0 software by Stat-Ease Inc. BBD is an experimental statistical design based on response surface methodology (RSM) that allows the effects and interactions of multiple variables to be evaluated using a relatively small number of experimental runs. This is an independent quadratic design that does not insert factorial and fractional factorial design. BBD uses relatively few continuous factors ≥ 3 [87,88]. In the present study, three independent variables which included amount of cholesterol (A), surfactant concentration (Span 60, B), and sonication time (C) were selected. The dependent variables were vesicle size (nm), entrapment efficiency (%), and drug release (%), as mentioned in Table 9.

All formulations contained lupeol as the active drug. The Box–Behnken Design generated the 15 experimental runs with 3 center points, and the experiments were conducted in a randomized order to minimize the systemic errors. The effects of independent variables and their interactions to the dependent variables were analyzed using different model fits in a sequential order, including the linear, 2FI, and quadratic models. Statistical analysis was performed using analysis of variance (ANOVA) where quality parameters were evaluated (*p* ≤ 0.05). Based on the statistical outcomes, most appropriate model was selected.

To ensure effective topical delivery, formulation criteria were set to target a 300 nm vesicle size [89,90], maintain entrapment efficiency between 66 and 91%, and maximize drug release. Because entrapment efficiency and drug release represent different formulation attributes, maximizing both responses (EE% and drug release) simultaneously may not necessarily be achievable [91]; therefore, EE was maintained within an acceptable range while drug release was maximized.

#### 4.2.5. Characterization of Lupeol-Loaded Bilosomes (LUPB)

##### Morphology

Transmission electron microscopy (TEM) was utilized to examine the morphology and structural characteristics of the lupeol-loaded bilosomes. A drop of the LUPB dispersion was carefully placed onto a carbon-coated copper grid and allowed to adsorb for a few minutes. The excess dispersion was gently removed using filter paper. The sample was then negatively stained with 1% (*w*/*v*) phosphotungstic acid to enhance contrast. After air-drying at room temperature for approximately 10 min, the prepared grid was examined using a transmission electron microscope to visualize the vesicular morphology [92].

##### Vesicle Size and Polydispersity Index (PDI)

The mean vesicle size, polydispersity index (PDI), and zeta potential of the prepared bilosomes were determined with the help of a zetasizer (Malvern Zetasizer, Malvern Instruments, Worcestershire, UK). A small quantity of bilosomal dispersion (10 µL) was diluted in distilled water/Milli-Q water (1 mL) to avoid the multiple scattering effect during the experiment. The vesicle size and surface charge were measured at 25 °C with the scattering angle of 90° (before dilution). For each experimental run generated by the Box–Behnken Design (BBD), a single formulation batch was prepared. Each batch was measured in triplicate (n = 3 technical replicates) on the same sample to ensure measurement repeatability. The mean values of these technical replicates were subsequently utilized as response inputs for statistical modeling and analysis in Design-Expert software [93,94].

##### Entrapment Efficiency (EE %)

Entrapment efficiency indicates the percentage of total drug encapsulated within the bilosomes. This was done by the cooling centrifugation method at 10,000 rpm for 30 min at 4 °C. The resultant sediment containing drug-loaded vesicles was disrupted by Triton X-100 (0.1%) and the sample was recentrifuged under the same conditions. The resultant clear aliquot was filtered using a 0.45 µm syringe filter, and the filtrate was analyzed spectrophotometrically (UV 1800 Shimadzu) at λmax 221 nm [94]. The entrapment efficiency (EE %) was calculated using the formula:

(4) $$\%\;\mathrm{EE}\;=\;\frac{Amount\;of\;drug\;entrapped}{Total\;amount\;of\;drug}$$

##### Preparation of Lupeol-Loaded Bilosomal Gel (LUPBG)

Base gel (1% *w*/*v*) was prepared by dispersing 1 g of Carbopol 934P in 100 mL distilled water. The formulations were assessed for gel formation, physical appearance, and viscosity. The results demonstrated that concentrations below 1.0% *w*/*w* produced gels with insufficient consistency, whereas 1.0% *w*/*w* produced a homogeneous and physically stable gel with satisfactory rheological properties Increasing the polymer concentration beyond 1.0% *w*/*w* resulted in increased viscosity and a corresponding reduction in spreadability. Therefore, 1.0% *w*/*w* Carbopol 934P was identified as the minimum gelation concentration (MGC) and selected as the minimum polymer concentration required to obtain a suitable gel system (Supplementary file). This selection is consistent with several published studies reporting that 1% Carbopol provides suitable gel characteristics, formulation performance, and drug-release properties [95,96]. The dispersion was stirred at 1000 rpm for 1 h at room temperature using a digital stirrer (HLS 200, LabQuest, Mumbai, India) [97]. The suspension was left overnight to allow complete hydration and swelling of the polymer. Methyl paraben (0.1%) and triethanolamine were added to adjust the pH to promote the gel’s viscosity. The optimized bilosomal dispersion was transferred into suitable glass vials and pre-freezed at −80 °C for 2 h. The frozen samples were subsequently subjected to lyophilization. Primary drying was carried out under vacuum at a shelf temperature of −20 °C and a chamber pressure of approximately 0.1 mbar for 24 h. Secondary drying was carried out at +20 °C under a pressure of approximately 0.1 mbar for 15 h to remove residual moisture. The lyophilized bilosomal formulation was then added into the base gel to obtain a 1% *w*/*w* homogeneous and viscous lupeol-loaded bilosomal (LUPBG) gel. The lupeol-loaded conventional gel (LUPCG) was prepared by adding the same amount of lupeol into pre-hydrated base gel under continuous stirring. The same concentration of carbopol, triethanolamine, and preservative was used for LUPCG and LUPBG, and the final weight of both formulations was adjusted to the same value.

#### 4.2.6. Characterization of Lupeol-Loaded Bilosomal Gel

##### pH, Viscosity and Spreadability

**Figure 17:**
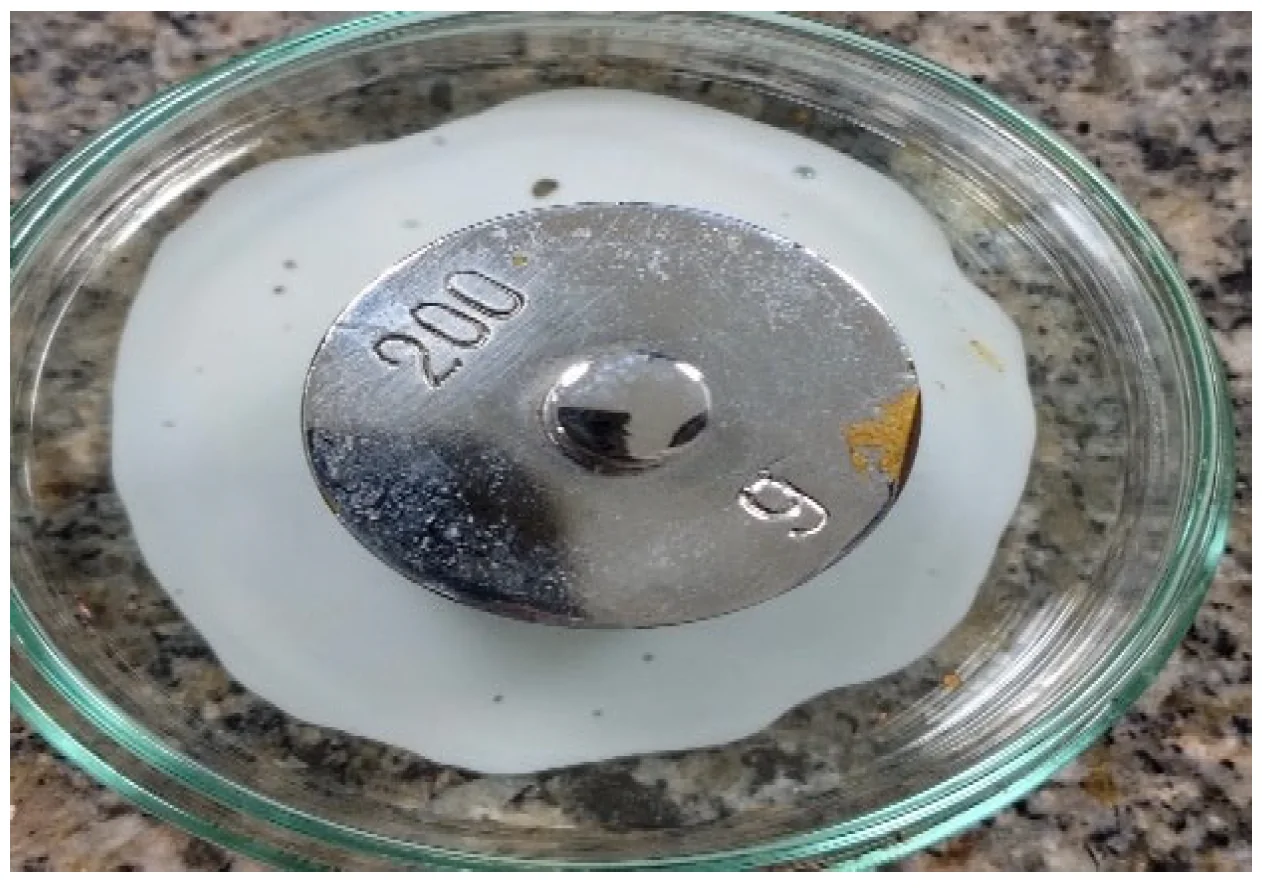
Spreadability of the optimized bilosomal gel (LUPBG).

The pH of the optimized LUPBG gel was measured with a digital pH meter (Labman pH meter, LMPH10H). The pH of the gel was measured by dipping the electrode for four minutes into the sample. The viscosity of LUPBG gel was determined by Brookfield digital viscometer (DV2T viscometer, AMETEK Brookfield, Middleboro, MA, USA). The study was performed by using spindle number 6 at 100 RPM at room temperature. The spreadability of LUPBG was analyzed by placing it on a clean Petri dish and initial diameter was measured. The second Petri dish was placed on the first one with a weight (200 g) for 30 s (Figure 17). The final diameter was measured and the spreadability was calculated by the given equation [98,99].

(5) $$\mathrm{Spreadability}\;(\%)=\frac{Final\;diameter-Initial\;diameter}{Initial\;diameter}\times 100$$

##### Drug Content

The drug content of the prepared LUPBG gel was determined by dissolving a known amount of the gel, 1 g (1 g gel equivalent to 10 mg lupeol), to 10 mL ethanol (10 mL). It was sonicated on bath sonicator for 15 min. After that the sample was filtered out using a 0.45 μm syringe filter and analyzed by UV spectrophotometer at 221 nm [94].

##### In Vitro Drug Release Study

**Figure 18:**
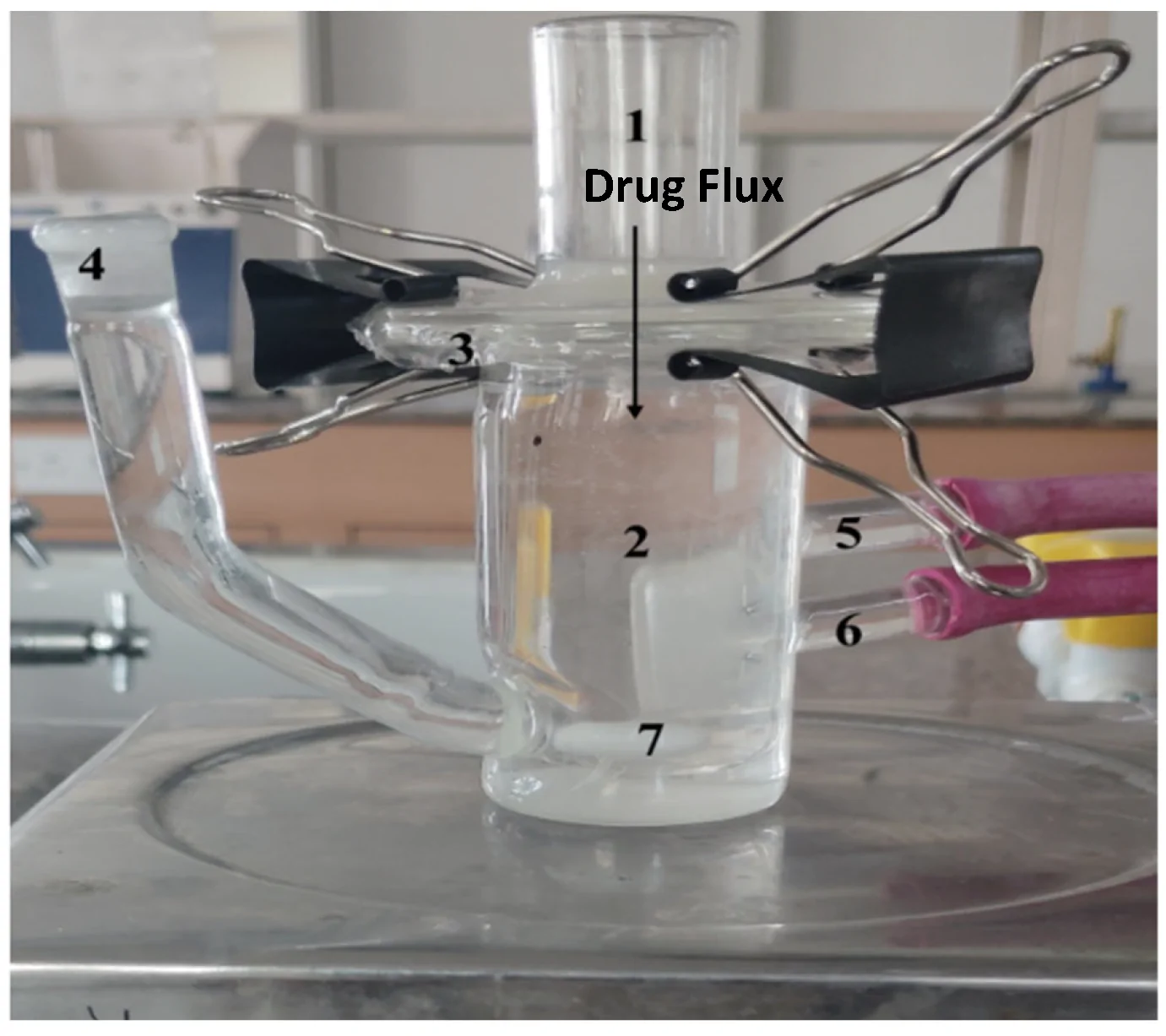
Franz diffusion cell (FDC) assembly for in vitro drug release study with dialysis membrane (12–14 kDa). (1) Donor chamber; (2) receptor chamber; (3) dialysis membrane; (4) sampling port; (5) water inlet; (6) water outlet; (7) magnetic bead; drug flux (1 to 2).

The in vitro release study was investigated using a dialysis membrane-assisted method in Franz diffusion cell (4.91 cm^2^ area and 35 mL volume). The samples of LUPB, LUPBG and LUPCG (equivalent to 10 mg) were placed into the donor compartment. The dialysis membrane (molecular weight cut-off between 12,000 and 14,000 Da) was placed between the donor and receptor compartments as shown in Figure 18. The membrane was soaked overnight in the release media before the experiment. Phosphate buffer (pH 6.8) containing 25% ethanol was used as the release medium to improve lupeol solubilization and support sink conditions [100]. The receptor compartment was continuously stirred at 100 RPM and maintained at a temperature of 37 ± 0.5 °C throughout the experiment. Samples (1 mL) were withdrawn at predefined intervals from the receptor chamber and an equal volume of ethanol–phosphate buffer (75:25) was replaced to maintain the sink condition throughout the experiment. The collected aliquots were filtered through a 0.45 µm syringe filter and analyzed by UV spectrophotometer in triplicate (n = 3). The release study was conducted for 24 h to observe the release profile [101]. Lupeol quantification was carried out at medium-specific maximum absorption wavelengths (λmax) to account for solvent-induced solvatochromic shifts. For the determination of entrapment efficiency, lupeol was extracted/diluted in ethanol (λmax = 221 nm), whereas for the in vitro release study, phosphate buffer (pH 6.8) was used as the release medium, and the λmax of lupeol in this medium was determined to be 211 nm. The use of medium-specific λmax values aligns with established analytical protocols for lupeol nanocarriers. Similar approaches are well-documented: Zhang et al. quantified lupeol entrapment in methanol at 210 nm, while Karunanidhi et al. evaluated release in pH 6.8 buffer at 211 nm [102,103].

The cumulative drug release data of the LUPBG formulation was fitted into different kinetic models including first-order, Korsmeyer–Peppas, and Higuchi kinetic models to understand the mechanism of drug release. The best-fitting model was identified based on the highest regression coefficient [48].

##### Texture Analysis

Mechanical properties like cohesiveness, adhesiveness and hardness of optimized bilosomal formulation were evaluated using a texture analyzer (Texture Pro CT V1.3 Build 15, Brookfield, AMETEK Inc., Middleboro, MA, USA) and Texture Profile Analysis (TPA) was chosen and the cone probe TA3/100 was used. The experimental conditions were set as follows: 10 kg load cell, 5.0 g trigger force, 1.5 mm/s test speed and 5 mm/s return speed.

An amount of 50 g of the optimum formulation gel was placed in a 100 mL glass beaker, and the surface was kept as smooth as possible to prevent air bubble entrapment. The gel sample was evaluated at room temperature to assess their consistency and texture properties [55,104].

#### 4.2.7. Ex Vivo Permeation Study

Ex vivo permeation of LUPBG was studied using a Franz diffusion cell and compared with 1% *w*/*w* LUPCG and LUPB. Ex Vivo skin permeation was carried out on freshly excised dorsal skin of Swiss albino mice. Skin was excised, washed, and kept in phosphate buffer pH 6.8. The skin was mounted between the donor and receptor cells of the Franz diffusion cell, and the remaining procedure is followed as mentioned previously in the in vitro release study [24].

The concentration of the drug that permeated through the skin was plotted against time. The steady state flux (Jss, μg/cm^2^/h) was calculated from the linear portion of the plot. The Permeability Coefficient (K_p_) of lupeol was calculated using the equation derived from Fick’s first law of diffusion as follows:

(6) $$K_{p}\;=\;\frac{J}{C}$$

#### 4.2.8. In Vivo Studies

##### Experimental Animals

The experimental protocols for pharmacological screening were thoroughly reviewed and approved by the Institutional Animal Ethical Committee (IAEC) of Amity Institute of Pharmacy, Amity University Uttar Pradesh, Lucknow. All of the procedures were determined to be in accordance with the rules established by the Committee for Control and Supervision of Experiments on Animals (CCSEA) under the Government of India (proposal number: AUUP/AIP/14/2024, approval date: 23 October 2024). Thirty male Swiss albino mice weighing between 25 and 30 g were used in the investigation. All animals were acclimatized for 7 days under conventional laboratory settings, including a temperature of 23 ± 2 °C, a 12 h light/dark cycle, and a relative humidity of 55 ± 5%. Throughout the study, the mice were provided with a standard rodent diet and had ad libitum access to water.

##### Experimental Design

Following the 1-week acclimation phase, the dorsal area of the mice was carefully shaved to create a hair-free area. Thirty male Swiss albino mice were randomly assigned to five different groups. Each group consisted of 6 mice (n = 6 each group). The experimental groups included a healthy control group (CL) and a disease control group (DCL), along with the standard drug tacrolimus (TAC) treatment group, the lupeol-loaded bilosomal gel (LUPBG) treatment group, and the lupeol-loaded conventional treatment group (LUPCG). The sample size was selected based on previously reported preclinical studies employing the imiquimod (IMQ)-induced psoriasis mouse model and considering the need to obtain sufficient biological replicates for statistical comparison while minimizing the number of animals used in accordance with the principles of the 3Rs (Replacement, Reduction, and Refinement). A recent study of an HDAC6 inhibitor used 30 mice divided into five groups, with six mice per group in an IMQ-induced psoriasis-like inflammation model [105,106].

##### Induction of Psoriasis and Treatment Phase

The therapeutic efficacy of lupeol-loaded bilosomal gel was evaluated using an imiquimod (IMQ)-induced plaque-type psoriasis model in thirty Swiss albino male mice. Psoriasis-like skin inflammation was induced by the topical application of 62.5 mg of 5% *w*/*w* IMQ cream on the shaved dorsal skin of the mice once daily for 7 consecutive days. In contrast, the CL group received a topical application of Vaseline instead of IMQ cream. The progression of psoriasis-like lesions was monitored daily.

Following successful disease induction, the treatment phase was initiated from day 8 and continued for 9 consecutive days (up to day 16). The TAC group received the standard drug tacrolimus, the LUPBG group was treated with lupeol-loaded bilosomal gel, and the LUPCG group was treated with lupeol-loaded conventional gel. The CL and DCL groups continued to receive vaseline and imiquimod cream (IMQ) throughout the study period [107,108].

#### 4.2.9. Evaluation Parameters of In Vivo Study

##### Psoriasis Area and Severity Index (PASI) Score

The PASI score was used to assess the severity of psoriatic lesions on days 2, 4, 6, 7, 10, 12, 14, and 16. Three core clinical features of psoriasis: skin erythema (redness), induration (thickness), and desquamation (scaling), were graded on a scale of 0–4 (none = 0, slight = 1, moderate = 2, marked = 3, and very marked = 4). The PASI score was calculated by summing the scores for erythema, desquamation and induration resulting in a possible range of 0–12, which enables quantitative assessment of both psoriasis severity and treatment response [109].

##### Spleen Index

The mice were first sacrificed, and then their spleens were excised and weighed [68]. Spleen index was calculated as

(7) $$\mathrm{Spleen}\;\mathrm{index}=\frac{Weight\;of\;spleen}{Body\;weight}\times 100$$

##### Complete Blood Counts (CBC)

Blood parameters were analyzed by performing a CBC using hematology analyzer (BC 5150, Mindray, Shenzhen, China). Whole blood samples were collected via cardiac puncture test into ethylene diamine tetra acetic acid (EDTA)-coated tubes. The blood samples were analyzed for red blood cells (RBCs), white blood cells (WBCs), hemoglobin, and platelets (PLT) count [110].

##### Evaluation of TNF-α Level

Diseased skin samples were collected from the mice from the DCL group to the LUPCG group, exhibiting psoriasis-like symptoms for the estimation of TNF- α levels. The excised skin tissues were homogenized using a tissue homogenizer to obtain a uniform skin homogenate. The homogenate was prepared in phosphate buffer (pH 7.4) and subsequently centrifuged at 3000 rpm for 30 min. The obtained supernatant was collected and stored at −4 °C until further biochemical analysis. The concentration of TNF-α was quantified using a commercially available ELISA kit according to the manufacturer’s protocols [107].

##### Histopathological Analysis

At the end of the experimental protocol, the mice were sacrificed, and dorsal skin samples were collected from each experimental group. The excised tissues were fixed in 10% *v*/*v* formaldehyde solution prepared in 0.1 M phosphate buffer (pH 7.4). Subsequently, the tissues were embedded in paraffin wax, and thin sections of approximately 6–8 μm thickness were prepared and mounted on adhesive glass slides. The tissue sections were stained with hematoxylin and eosin (H & E) to evaluate the skin histoarchitecture. Histopathological examinations were carried out under a light microscope at 100× and 200× magnification to evaluate epidermal thickness, and inflammatory cells infiltration [111].

#### 4.2.10. Statistical Analysis

All experimental data was analyzed by the GraphPad Prism 5 software. The results are expressed as mean ± SEM (n = number of samples per group). A two-way analysis of variance (ANOVA) was used to determine significant differences in PASI and drug release parameters followed by the Bonferroni multiple comparison test because these responses were evaluated across two independent factors (mice group and time). For the rest of the parameters, one-way ANOVA was used followed by Tukey’s multiple comparison test at a single time point to examine intergroup differences. *p*-values < 0.05 indicated statistical significance.

## Figures and Tables

**Table 1:** Composition of lupeol-loaded bilosomes and their corresponding characterization results. All formulations were analysed in triplicate (n = 3), and the data are presented as mean ± S.D.

Formulation Code	Factor 1	Factor 2	Factor 3	Response 1	Response 2	Response 3
	A: Cholesterol (mg)	B: Span 60 (%)	C: Sonication (min)	Vesicle Size (nm)	Entrapment Efficiency (%)	Drug Release (%)
F1	10.00	50.00	15.00	217 ± 0.23	74 ± 0.10	75 ± 0.32
F2	30.00	50.00	15.00	305 ± 0.33	79 ± 0.35	58 ± 0.05
F3	10.00	70.00	15.00	260 ± 0.09	76 ± 0.17	90 ± 0.78
F4	30.00	70.00	15.00	224.8 ± 0.6	91 ± 0.07	76 ± 0.62
F5	10.00	60.00	10.00	270 ± 0.54	69 ± 0.06	88 ± 0.08
F6	30.00	60.00	10.00	390 ± 0.21	85 ± 0.47	75 ± 0.45
F7	10.00	60.00	20.00	305 ± 0.19	66 ± 0.36	95 ± 0.18
F8	30.00	60.00	20.00	222 ± 0.21	73 ± 0.41	81 ± 0.71
F9	20.00	50.00	10.00	320 ± 0.07	85 ± 0.29	55 ± 0.15
F10	20.00	70.00	10.00	301 ± 0.45	88 ± 0.18	67 ± 0.23
F11	20.00	50.00	20.00	262 ± 0.13	72 ± 0.07	60 ± 0.72
F12	20.00	70.00	20.00	240 ± 0.06	86 ± 0.16	78 ± 0.18
F13	20.00	60.00	15.00	285 ± 0.03	83 ± 0.28	72 ± 0.71
F14	20.00	60.00	15.00	288 ± 0.02	83 ± 0.34	70 ± 0.43
F15	20.00	60.00	15.00	285 ± 0.17	85 ± 0.21	71 ± 0.23

**Footnote:** n = 3 represents technical replicates (three repeat measurements performed on a single formulation batch) to assess instrumental precision rather than batch-to-batch variability. Values are expressed as mean ± S.D.

**Table 2:** Model comparison statistics for responses vesicle size (nm), entrapment efficiency (%), and drug release (%).

Response	Vesicle Size (nm)			Entrapment Efficiency			Drug Release (%)		
Model/ Statistics	Linear	2FI	Quadratic	Linear	2FI	Quadratic	Linear	2FI	Quadratic
PRESS	39,558.05	22,972.49	784.83	659.77	1152.10	42.00	1754.31	4249.61	45
−2LL	149.38	127.98	64.74	89.01	85.13	25.84	102.83	102.62	34.48
BIC	160.21	146.94	89.11	99.84	104.09	52.92	113.66	121.57	56.15
AICc	161.38	157.98	118.74	101.01	115.13	100.84	114.83	132.62	74.48

PRESS, Predicted Residual Error Sum of Squares; BIC, Bayesian Information Criteria; AICc, Akaike’s Information Criteria; −2LL, −2 Log Likelihood.

**Table 3:** The regression analysis for selecting the best-fit model for vesicle size (nm), entrapment efficiency (%) and drug release (%).

Response (Unit)	Regression Parameters	Models			Best-Fitted Model
		Linear	2FI	Quadratic	
Vesicle size (nm)	SD	41.07	23.60	3.56	Quadratic model
	R^2^	0.3435	0.8423	0.9978	
	Adjusted R^2^	0.1645	0.7241	0.9946	
	Predicted R^2^	−0.3995	0.1873	0.9722	
	Adequate precision	4.0288	8.48	66.5953	
	% CV	14.76	10.2018	1.19	
	*p*-value	0.1851	0.0074	<0.0001	
Entrapment efficiency (%)	SD	5.49	5.66	0.9916	Quadratic model
	R^2^	0.5831	0.6780	0.9938	
	Adjusted R^2^	0.4694	0.4365	0.9827	
	Predicted R^2^	0.1704	−0.4486	0.9472	
	% CV	6.89	7.10	1.24	
	Adequate precision	6.5251	5.7245	31.34	
	*p*-value	0.0184	0.5345	<0.0001	
Drug release (%)	SD	8.70	10.13	1.12	Quadratic model
	R^2^	0.5508	0.5570	0.9953	
	Adjusted R^2^	0.4283	0.2248	0.9906	
	Predicted R^2^	0.0542	−1.2910	0.9757	
	% CV	11.75	13.68	1.51	
	Adequate precision	6.7308	4.3693	49.7553	
	*p*-value	0.0272	0.9896	<0.0001	

**Table 4:** The reduced analysis of variance (ANOVA) for the quadratic response surface models of vesicle size (nm), entrapment efficiency (%) and drug release (%).

Vesicle Size (nm)					
Source	Sum of the Squares	DF	Mean Square	F-Value	*p*-Value
Model	28,200.94	8	3525.12	321.63	<0.0001 *
A: Cholesterol	1008.01	1	1008.01	91.97	<0.0001 *
B: Span 60	764.41	1	764.41	69.74	0.0002 *
C: Sonication	7938.00	1	7938.00	724.27	<0.0001 *
AB	3794.56	1	3794.56	346.22	<0.0001 *
AC	10,302.25	1	10,302.25	939.99	<0.0001 *
A^2^	309.13	1	309.13	28.21	0.0018 *
B^2^	2335.47	1	2335.47	213.09	<0.0001 *
C^2^	1462.19	1	1462.19	133.41	<0.0001 *
Residual	65.76	6	10.96	-	-
Lack of Fit	59.76	4	14.94	4.98	0.1742 **
Pure Error	6.00	2	3.00	-	-
Total	28,266.70	14	-	-	-
**Entrapment Efficiency (%)**					
Model	790.42	9	87.82	89.31	<0.0001 *
A: Cholesterol	231.12	1	231.12	235.04	<0.0001 *
B: Span 60	120.12	1	120.12	122.16	0.0001 *
C: Sonication	112.50	1	112.50	114.41	0.0001 *
AB	25.00	1	25.00	25.42	0.0040 *
AC	20.25	1	20.25	20.59	0.0062 *
BC	30.25	1	30.25	30.76	0.0026 *
A^2^	160.03	1	160.03	162.74	<0.0001 *
B^2^	31.41	1	31.41	31.94	0.0024 *
C^2^	54.26	1	54.26	55.18	0.0007 *
Residual	4.92	5	0.9833	-	-
Lack of Fit	2.25	3	0.75	0.5625	0.6904 **
Pure Error	2.67	2	1.33	-	-
Total	795.33	14	-	-	-
**Drug Release (%)**					
Model	1846.18	7	263.74	210.99	<0.0001 *
A: Cholesterol	420.50	1	420.50	336.40	<0.0001 *
B: Span 60	496.12	1	496.12	396.90	<0.0001 *
C: Sonication	105.13	1	105.13	84.10	<0.0001 *
BC	9.00	1	9.00	7.20	0.0314 *
A^2^	509.77	1	509.77	407.82	<0.0001 *
B^2^	236.31	1	236.31	189.05	<0.0001 *
C^2^	14.77	1	14.77	11.82	0.0109 *
Residual	8.74	7	1.25	-	-
Lack of Fit	6.75	5	1.35	1.35	0.4773 **
Pure Error	2.00	2	1.00	-	-
Total	1854.93	14	-	-	-

* Significant *p* < 0.0001, ** non-significant *p* > 0.05.

**Table 5:** Numerical optimization results for validation of the optimized formulation.

A: Cholesterol (mg)	B: Surfactant (Span 60) (%)	C: Sonication (min)	Vesicle Size (nm)	Entrapment Efficiency (%)	Drug Release (%)	Desirability	
30 mg	63.535%	13.344 min	300	86.321	76.128	0.820	Selected

**Table 6:** The comparison between the predicted and observed values of the dependent variables (responses) for the optimized lupeol-loaded bilosomes.

Response	Predicted Mean	Observed Mean	Standard Deviation	% Accuracy	95% PI Lower	95% PI Upper
Y_1_: Vesicle Size (nm)	300.013	300.9	±3.31059	99.70%	290.348	309.678
Y_2_: % EE	86.3211	88	±0.991623	98.05%	83.2762	89.366
Y_3_: % DR	76.3211	84.56	±1.11803	89.20%	73.0349	79.2197

**Table 7:** In vitro drug release kinetics of lupeol from lupeol-loaded bilosomal gel (LUPBG).

Formulation	Zero-Order (R2)	First Order (R2)	Higuchi (R2)	Hixon–Corwell (R2)	Korsmeyer–Peppas (R2, n)
LUPBG	0.8383	0.9219	0.9922	0.9886	0.9761, 0.558

**Table 8:** Effect of different treatments on blood parameters in experimental animals (n = 6).

Parameters	CL	DCL	LUPBG	LUPCG	TAC	Range
Haemoglobin (g/dL)	13.6 ± 0.25	10.2 ± 0.34	12.9 ± 0.27	12.1 ± 0.3	13.3 ± 0.24	12–16
Neutrophil (%)	48 ± 2	67.0 ± 1.62	53.0 ± 1.3	56.0 ± 1.7	50.0 ± 2	21–57
Lymphocytes (%)	46 ± 2	28.0 ± 1.79	42.0 ± 1.8	38.0 ± 2.1	45.0 ± 2	49–82
Eosinophil (%)	4 ± 0.5	9.0 ± 0.7	5.0 ± 0.48	6.0 ± 0.5	4.0 ± 0.40	0–4
Monocytes (%)	3 ± 0.40	8.0 ± 0.27	4.0 ± 0.3	5.0 ± 0.36	3.0 ± 0.3	2–8
RBC count (Million/Cumm)	7.1 ± 0.20	5.2 ± 0.16	6.8 ± 0.19	6.3 ± 0.18	6.4 ± 0.31	7–12.5
Platelet count (Lac/Cumm)	427 ± 2	710 ± 1.3	421 ± 2.1	650 ± 2.5	490 ± 1.7	418–626
NLR (Neutrophil-to-Lymphocyte ratio)	1.04	2.39	1.26	1.47	1.11	
LMR (Lymphocyte-to-Monocyte ratio)	15.33	3.5	10.5	7.6	15	

**Table 9:** Box–Behnken design for the optimization of lupeol-loaded bilosomes including independent variables and response constraints.

Independent Variables	Levels		
	(−1)	(0)	(+1)
A: Amount of cholesterol (mg)	10	20	30
B: Surfactant concentration (%)	50	60	70
C: Sonication time (minute)	10	15	20
Responses	Desirability constraints		
Y_1_: Vesicle size	Set to target = 300 nm		
Y_2_: %Entrapment efficiency	Is in range (66–91%)		
Y_3_: %Drug release (24 h)	Maximum		

## Data Availability

Study data are available from Amity Institute of Pharmacy, Amity University, Lucknow Campus.

## Supplementary Materials

The following supporting information can be downloaded at: https://www.mdpi.com/article/10.3390/gels12100918/s1, Table S1. Optimization of the Carbopol 934P Gel Base at Different Polymer Concentrations. The table presents the observations, spreadability, and viscosity of formulations containing 0.50%, 0.75%, 1.00%, 1.25%, and 1.50% (*w*/*w*) Carbopol 934P.

## Abbreviations

The following abbreviations are used in this manuscript:

(TNF)-α	Tumor necrosis factor α
BCS	Biopharmaceutics classification system
ANOVA	Analysis of variance
PDA	photodiode array
TEM	Transmission electron microscopy
ELISA	Enzyme-linked immunosorbent assay
PASI	Psoriasis area and severity index
