# Supplementary material for: Development and Optimization of a Lupeol-Loaded Bilosomal Gel for Enhanced Topical Therapy of Imiquimod-Induced Psoriasis
Source: Gels. 2026 Oct 13;12(10):918. doi: 10.3390/gels12100918 (PMC13704759; doi:10.3390/gels12100918)
Supplement: Supplementary file 1 [file gels-12-00918-s001.zip › gels-4553733-supplementary.pdf]

Manuscript ID: gels-4553733

Title: Development and Optimization of a Lupeol-Loaded Bilosomal Gel for Enhanced Topical Therapy of Imiquimod-Induced Psoriasis

In drug release studies, it is generally desirable to minimize the amount of polymeric gelling agents used as excipients in the formulation, in order to reduce their potential influence on the drug release behavior. Therefore, I recommend that the authors determine the minimum gelation concentration (MGC) of the developed drug-loaded gel system. Such an evaluation would help optimize the amount of gelling agent used and provide stronger evidence regarding the suitability and formulation efficiency of the developed system.

Table S1: Optimization of base carbopol 934P gel

**Formulation**	**Carbopol concentration (% w/w)**	**Observation**	**Spreadability**	**Viscosity**
F1	0.50%	Weak gel, poor consistency	43 g.cm/s	2220 cP
F2	0.75%	Gel formed but inadequate consistency	37 g.cm/s	3551cP
**F3**	**1.00%**	**Stable, homogeneous gel with acceptable consistency**	**33 g.cm/s**	**5919 cP**
F4	1.25%	Very viscous; may retard drug release	23 g.cm/s	7870 cP
F6	1.50%	Excessively viscous; undesirable for topical application	18 g.cm/s	9891 cP
